# Efficacy and safety of biological agents for the treatment of pediatric patients with psoriasis: A bayesian analysis of six high-quality randomized controlled trials

**DOI:** 10.3389/fimmu.2022.896550

**Published:** 2022-08-19

**Authors:** Xiao-ce Cai, Yi Ru, Liu Liu, Xiao-ying Sun, Ya-qiong Zhou, Ying Luo, Jia-le Chen, Miao Zhang, Chun-xiao Wang, Bin Li, Xin Li

**Affiliations:** ^1^ Department of Dermatology, Yueyang Hospital of Integrated Traditional Chinese and Western Medicine, Shanghai University of Traditional Chinese Medicine, Shanghai, China; ^2^ Institute of Dermatology, Shanghai Academy of Traditional Chinese Medicine, Shanghai, China; ^3^ Shanghai Skin Disease Hospital, School of Medicine, Tongji University, Shanghai, China

**Keywords:** biological agents, pediatric, psoriasis, adverse events, Bayesian analysis, systematic review

## Abstract

**Background:**

Biological agents have been used with extreme caution in children because of their possible adverse effects.

**Objectives:**

This study used high-quality randomized controlled trials (RCTs) to provide high-level evidence to assess the effectiveness and safety of biological agents for treating children with psoriasis.

**Methods:**

We searched PubMed, Embase, Cochrane, and Web of Science databases through October 31, 2021. We included trials reporting at least one adverse event after treatment with biological agents of patients less than 18-year-old diagnosed with psoriasis. RevMan 5.3 and Stata 15.0 software were used for meta and Bayesian analyses.

**Results:**

Six trials with 864 participants were included in the analysis. The results showed a 2.37-fold higher response rate in all biologics groups than in the control group for psoriasis area and severity index 75 (PASI75) (RR= 2.37, *P-*value < 0.01, 95% confidence interval [CI] [1.22, 4.62]). Compared with placebo, the PASI75 response rates of etanercept (RR= 2.82, 95% [CI] [1.10, 7.21]), ustekinumab low dose (RR= 7.45, 95%[CI] [1.25, 44.58]), and ustekinumab high dose (RR= 7.25, 95%[CI] [1.21, 43.41]) were superior. Additionally, the incidence of total adverse reactions was 1.05 times higher for biologics than for controls, indicating a good safety profile (RR= 1.05, *P-*value = 0.53, 95%[CI] [0.92, 1.19]). Overall, these six high-quality randomized controlled trials suggest that biologics are effective and safe for pediatric patients with psoriasis.

**Limitations:**

Inclusion of few relevant, high-quality RCTs.

**Conclusion:**

The results of this study indicate that biologics can be used to treat children with moderate-to-severe psoriasis without the risk of adverse effects. Ustekinumab showed the best efficacy and the fewest adverse effects.

## Introduction

Psoriasis is a common immune-mediated condition primarily characterized by skin lesions, affecting approximately 3% of the population worldwide (1), and nearly one-third of the patients present with disease symptoms before adulthood (2). Pediatric psoriasis is often highly visible and uncomfortable, and approximately 20% of children present with moderate to severe disease, which may require effective systemic therapy (3). Biologics are an important therapeutic option for moderate-to-severe psoriasis when other treatments are contraindicated or ineffective (4). Evidence confirms that the correlation between biologics and serious infections is small in the general population; however, biologics have been used in special populations, such as children, pregnant women, and the elderly, with extreme caution owing to the risk of adverse effects (5).

In 2002, etanercept was approved by the US Food and Drug Administration (FDA) to treat psoriasis. Twelve years later, in 2014, it was expanded to the treatment of pediatric patients (4–17 years old) with chronic moderate-to-severe plaque psoriasis (6). In 2021, secukinumab was approved by the FDA for the treatment of patients aged 6–18 years with moderate-to-severe plaque psoriasis. Currently, ixekizumab and ustekinumab are also approved to treat patients over six years of age with mild-to-moderate plaque psoriasis. However, relevant evaluations for utilizing biologics in childhood and adolescent psoriasis are lacking.

Biological agents are engineered monoclonal antibodies and fusion proteins capable of therapeutic action by blocking specific cytokines or cytokine receptors critical to psoriatic inflammation (7). It is speculated that T_H_1 and T_H_17 mediate psoriasis, and inhibition of T_H_17 is a known therapeutic strategy. Drugs targeting tumor necrosis factor(TNF)-α, interleukin(IL)-23, and IL-17 are effective for the clinical treatment of psoriasis (8). TNF-α inhibitors dominate the therapeutic market for psoriasis treatment. Furthermore, IL-12/IL-23 and IL-17 inhibitors could reduce the expansion and production of T_H_17, thereby suppressing the production of pro-inflammatory factors with relatively few adverse reactions (9). Accordingly, biologics are being progressively used to rapidly and effectively decrease psoriasis severity, although side effects have been noted, along with relapses after drug withdrawal.

Several studies have demonstrated the relative safety of biologics in special populations, particularly pediatric and pregnant patients (10–12). However, individual reports have limited power to characterize the risk factors for AEs in children and adolescents, and clinicians and patients are eager to receive corresponding guidance. Therefore, this review collates information from currently available studies documenting the apparent side effects of biologics, which can direct future clinical trials.

## Methods

This review was conducted using the Cochrane Handbook on Systematic Review of Interventions and presented following the Preferred Reporting Items for Systematic Review and Meta-Analysis (PRISMA) guidelines (13) (**Data Sheet 1**).

### Search strategy

Four databases (PubMed, Embase, Cochrane Library, and Web of Science) were searched from inception to October 31, 2021. We combined subject headings and free text words to retrieve all relevant studies. The following keywords were used: (“clinic” OR “clinical”) and (“Secukinumab” OR “Brodalumab” OR “Ixekizumab” OR “Ustekinumab” OR “Guselkumab” OR “Tildrakizumab” OR “Risankizumab” OR “Brazikumab” OR “Mirikizumab” OR “Etanercept” OR “Adalimumab” OR “Infliximab”) and (“children” OR “adolescent” OR “teenager” OR “young” OR “boy” OR “girl” OR “pediatric”) and (psoriasis).

### Data inclusion

We determined the inclusion and exclusion criteria for the present analysis before conducting a literature search. The inclusion criteria were as follows: RCTs reporting at least one type of AE after intervention with biologics, subjects diagnosed with psoriasis and aged<18 years, regardless of sex and ethnicity.

### Data extraction

Two investigators (X.C. Cai and M. Zhang) independently screened the studies according to the inclusion criteria using self-designed data extraction templates for each included study. Two authors (X.C. Cai and X.Y. Sun) assessed the risk of bias, and three authors (Y.Q. Zhou, Y. Luo, and J.L. Chen) performed data analysis and interpretation.

### Outcome measures

The primary efficacy measure was the PASI 75, a quantitative rating score that measures the severity of psoriatic lesions based on area coverage and plaque appearance, including scaling, infiltration, and erythema. In addition, the PASI 50, PASI 90, PASI 100, sPGA, sPGA0/1, and DLQI 0/1 indices were used to evaluate the efficacy outcomes. The main outcome indicators for safety evaluations were AEs and serious AEs. The secondary indicators of adverse reactions were the five types of adverse reactions produced by biologics (14). This classification could help better deal with the clinical features of these side effects, identify individual and general risk factors, and direct research in this novel area of medicine.

### Statistical analysis

RevMan5.3 software provided by the Cochrane Collaboration, was used to synthesize the results of the meta-analysis. Risk ratios (RR) with 95% confidence intervals (CIs) were calculated for dichotomous data. The mean difference (MD) and standard mean difference (SMD) were used for continuous data. Across trials, a fixed-effects model was used for homogeneity (I^2^ < 25%); otherwise, a random-effects model was used. Statistical significance was set at *P-*value < 0.05.

Network analysis was conducted in Stata 15.0 and using Markov Chain Monte Carlo (MCMC) simulation random-effects modeling in a Bayesian frame. For each analysis, we used three chains and generated 100,000 iterations. The first 20,000 iterations were the burn-in periods to eliminate the effect of initial values, and the last 80,000 iterations were used for sampling. The convergence was evaluated using a trace plot. Inconsistency between direct and indirect comparisons was assessed by the “node-splitting” method. The surface under the cumulative ranking (SUCRA) (%) was calculated. If SUCRA approaches 100%, the intervention is the best among the included trials; conversely, if it is close to 0%, it represents the worst intervention.

## Results

### Study selection and characteristics

**Figure 1:**
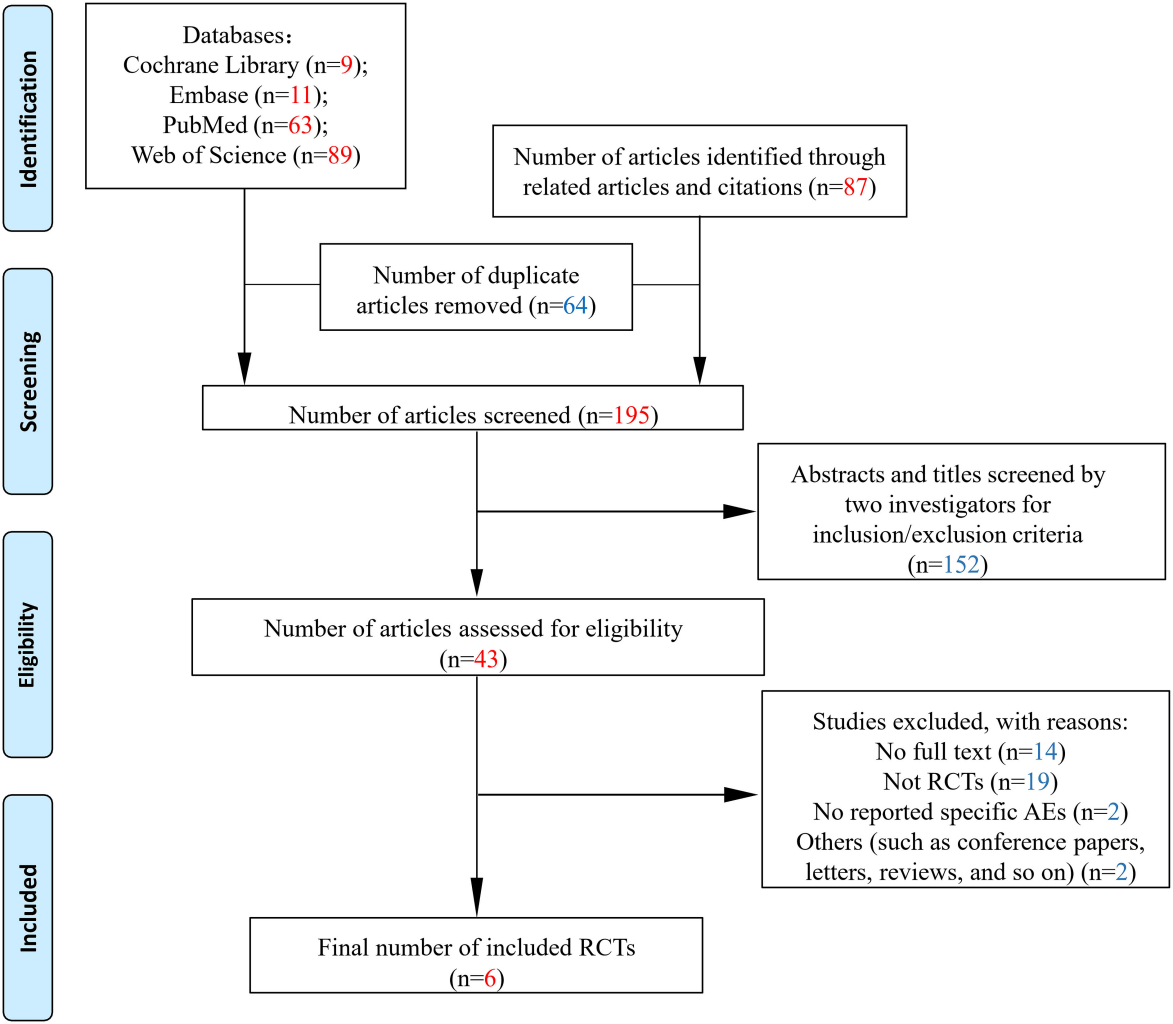
Flowchart of study inclusion according to the PRISMA 2009 guidelines. RCTs: randomized control trials; AEs, adverse events.

We identified 172 studies after a preliminary search of four databases and 87 related articles (related articles and citations). After removing duplicate studies, 195 remained. Subsequently, the titles and abstracts of each study were screened, and 43 studies were available for a detailed evaluation. Of the remaining articles, 37 were excluded, of which 14 were not available in full text, 19 were non-RCTs, 2 did not report AEs, and 2 were excluded due to other reasons such as the articles being letters. Six full-text articles were included in the final analysis (15–20). All the trials were published in English (**Figure 1**).

Finally, six trials involving 864 participants were included in the analysis: four clinical studies compared biologics with placebo, one study compared two biologics, and one compared a biologic with methotrexate (MTX) (**Table 1**).

Table 1Summary of included trials characteristics.

**Table:** 

Author year	Phase	Multicenter (Y/N)	Region	Enrollment number	Age limit	Severity			Sample size (M/F)				Male n%
									I		C		
**Bodemer 2021 (** 15)	III	Y	Global	NCT02471144	6 to <18	severe			30/50		16/25		39.0
**Paller 2020 (** 18)	III	Y	Europe and America	NCT03073200	6 to < 18	moderate-to-severe			52/63		20/36		42.2
**Papp 2017 (** 17)	III	Y	South America, North America, and Europe	NCT01251614	≥4 to <18	severe			38/39		11/26		43.0
**Landells 2015 (** 16)	III	Y	North America	NCT01090427	12 to 17	moderate-to-severe			20/17		34/39		49.1
**Siegfried 2010 (** 20)	III	Y	American	NCT00078819	4 to 17	moderate-to-severe			32/37		33/36		49.3
**Paller 2008 (** 19)	III	Y	American	NCT00078819	4 to 17	moderate-to-severe			55/51		53/52		51.2

Table 1 Continued.

**Table:** 

Author year	Sample age (yr)			Duration of treatment (wk)	Disease duration, yr				Target		Biologics		Control
	I Mean (SD)		C Mean (SD)		I Mean (SD)	C Mean (SD)							
**Bodemer 2021 (** 15)	13.5 (3.07)		13.5 (2.94)	52	5.15 (4.48)	4.55 (3.73)			IL-17A		Secukinumab		Etanercept
**Paller 2020 (** 18)	13.7 (4.14)		13.1 (2.79)	12	4.7 (3.26)	4.7 (3.01)			IL-17A		Ixekizumab		placebo
**Papp 2017 (** 17)	12.8 (4.0)		13.4 (3.5)	16	4.9 (3.6)	5.1 (3.8)			TNF-α		Adalimumab		MTX
**Landells 2015 (** 16)	14.9 (1.7)		15.6 (1.5)	12	5.7 (3.9)	6.2 (5.0)			IL-23		Ustekinumab		placebo
**Siegfried 2010 (** 20)	13.0		13.0	12	5.3	5.9			TNF-α		Etanercept		Placebo
**Paller 2008 (** 19)	14.0		13.0	12	6.8	5.8			TNF-α		Etanercept		placebo

Table 1 Continued.

**Table:** 

Author Year	Dose			Frequent		PASI 50	PASI 75	PASI 90	PASI100	SPGA 0/1	SPGA 0	CDQLI 0/1	AE
	I	C	I	C
**Bodemer 2021 (** 15)	75/150/300 mg		75/150/300 mg	0,1,2,3,4,then Q4W	0,1,2,3,4,then Q4W		●	●	●			●	●
**Paller 2020 (** 18)	0.8 mg/kg		/	Q4W	Q4W	●	●	●	●	●	●	●	●
**Papp 2017 (** 17)	0.8 or 0.4 mg/kg		0.1–0.4 mg/kg	Q2W	Q1W		●	●	●	●			●
**Landells 2015 (** 16)	0.75 or 0.375 mg/kg		/	weeks 0 and 4	weeks 0 and 4		●	●		●	●	●	●
**Siegfried 2010 (** 20)	0.8 mg/kg		/	Q1W	Q1W		●			●			●
**Paller 2008 (** 19)	0.8 mg/kg		/	Q1W	Q1W	●	●	●		●			●

Wk, week; y, year; M, male; F, female; Y, yes; N, no; I, intervention; C, control; SD, standard difference; AE, adverse event; PASI, Psoriasis Area and Severity Index; PASI 50/75/90/100, PASI score decreased by more than 50%, 75%, 90%, 100% from baseline; sPGA0/1, static physician’s global assessment achieve 0 or 1; sPGA0, static physician’s global assessment achieve 0; DLQI 0/1, Children’s Dermatology Life Quality Index achieved 0 or 1; MTX, methotrexate; Q1W, every 1 week; Q2W, every 2 weeks; Q4W, every 4 weeks; IL17A, interleukin-17A; Il-23, interleukin-23; TNF-α, tumor necrosis factor-α.

### Risk of bias assessment

**Figure 2:**
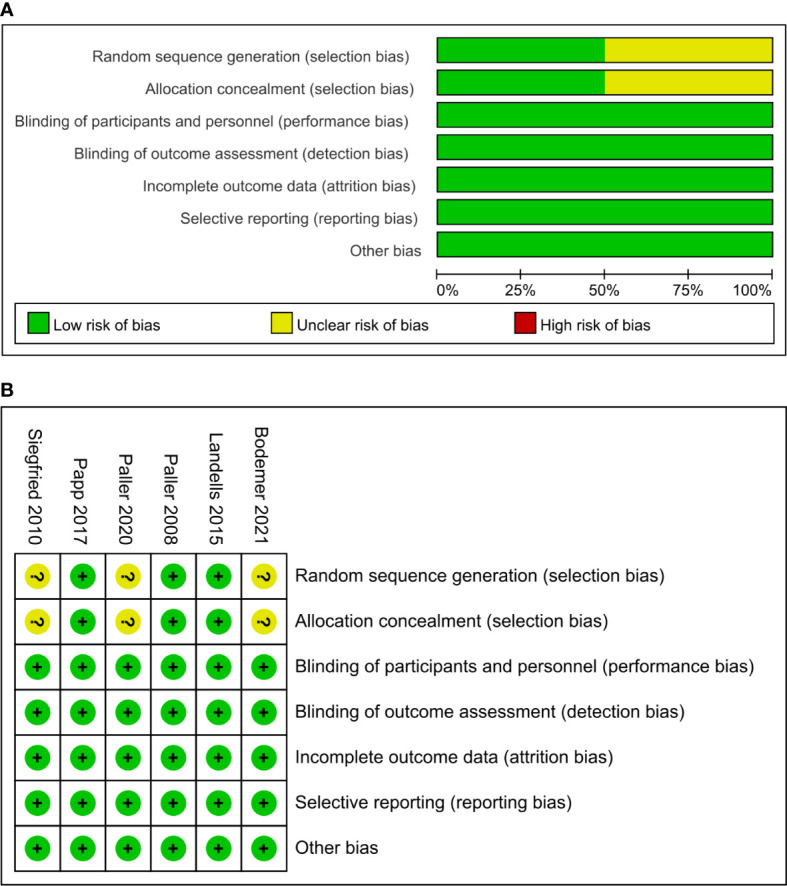
Risk of bias summary of clinical studies **(A)**. Risk of bias graph of clinical studies **(B)**.

All the included RCTs described a specific stochastic approach. Three studies (15, 18, 20) did not report details regarding selection bias. All studies were double-blinded (participants and personnel) and were assessed in a blinded manner. We evaluated the completed data without selective reporting or other bias. In summary, the quality of the included studies was high, and the results of this meta-analysis were credible (**Figure 2**).

### Outcomes

#### Efficacy

##### Psoriasis Area and Severity Index (PASI) 75

PASI 75 indicates that the lesion score improved by 75% compared with the baseline score. In the present study, the PASI 75 was used to evaluate the primary efficacy of biologics for broader applications. We observed that the efficacy of biologics (ustekinumab, ixekizumab, and etanercept) was significantly higher than that of placebo (**Table 2**, **Data Sheet 2**). In addition, adalimumab was more effective than MTX (**Table 2**; **Data Sheet 2**). Collectively, the biologic-treated groups presented 2.37-fold better results than the control group (**Table 2**, **Data Sheet 2**; *P-*value < 0.01, RR = 2.37, 95%[CI] [1.22, 4.62]).

**Table 2:** Efficacy of biological agents in pediatric patients with psoriasis.

Subgroup	Experimental		Control		Risk ratio M-H (95% CI)	Study weight, %	I^2^, %	P-value
	Events	Total	Events	Total				
**PASI 75**								
Ustekinumab vs. Placebo	58	73	4	37	7.35 [2.89,18.68]	13.6		0.01^#^
Ixekizumab vs. Placebo	102	115	14	56	3.55 [2.24,5.61]	17.0		0.01^#^
Etanercept vs. Placebo	112	174	67	174	2.15 [0.26,18.04]	34.6	98	0.01^#^
Secukinumab vs. Etanercept	72	80	28	41	1.32 [1.06,1.64]	18.1		0.01^#^
Adalimumab vs. MTX	39	77	12	37	1.56 [0.93,2.61]	16.7		0.09
Total(95% CI)	383	519	125	345	2.37 [1.22,4.62]	100.0	95	0.01^#^
**PASI 50**								
Etanercept vs. Placebo	79	106	24	105	3.26 [2.26,4.71]	46.1		0.01^#^
Ixekizumab vs. Placebo	106	115	21	56	2.46 [1.75,3.46]	53.9		0.01^#^
Total(95% CI)	185	221	45	161	2.83 [2.20,3.63]	100.0	18	0.27
**PASI 90**								
Ustekinumab vs. Placebo	42	73	2	37	10.64 [2.73,41.56]	16.5		0.01^#^
Ixekizumab vs. Placebo	90	115	3	56	14.61 [4.84,44.11]	18.4		0.01^#^
Etanercept vs. Placebo	29	106	7	105	4.10 [1.88,8.95]	20.7		0.01^#^
Secukinumab vs. Etanercept	64	80	21	41	1.56 [1.14,2.15]	23.1		0.01^#^
Adalimumab vs. MTX	23	77	8	37	1.38 [0.68,2.79]	21.2		0.37
Total (95% CI)	248	451	41	276	3.85 [1.40,10.58]	100.0	89	0.01^#^
**PASI 100**								
Ixekizumab vs. Placebo	57	115	1	56	27.76 [3.94,195.31]	29.9		0.01^#^
Secukinumab vs. Etanercept	36	80	9	41	2.05 [1.10,3.83]	40.8		0.02^*^
Adalimumab vs. MTX	11	77	1	37	5.29 [0.71,39.42]	29.3		0.10
Total (95% CI)	104	272	11	134	5.89 [0.84,41.51]	100.0	80	0.01^#^
**sPGA 0/1**								
Ustekinumab vs. Placebo	50	73	2	37	12.67 [3.26,49.22]	15.2		0.01^#^
Ixekizumab vs. Placebo	93	115	6	56	7.55 [3.53,16.16]	19.7		0.01^#^
Etanercept vs. Placebo	56	106	14	105	3.96 [2.36,6.66]	21.3	95	0.01^#^
Adalimumab vs. MTX	39	77	15	37	1.25 [0.80,1.96]	21.6		0.33
Total (95% CI)	276	439	27	304	3.15 [1.26,7.86]	100.0	93	0.01^#^
**sPGA 0**								
Ustekinumab vs. Placebo	29	73	1	37	14.70 [2.08,103.71]	49.7		0.01^#^
Ixekizumab vs. Placebo	60	115	1	56	29.22 [4.16,205.41]	50.3		0.01^#^
Total (95% CI)	89	188	2	93	22.01 [5.53,87.60]	100.0	0	0.62
**CDLQI 0/1**								
Ustekinumab vs. Placebo	29	73	4	37	3.67 [1.40,9.67]	22.6		0.01^#^
Ixekizumab vs. Placebo	74	115	13	56	2.77 [1.69,4.55]	37.6		0.01^#^
Secukinumab vs. Etanercept	42	80	16	41	1.35 [0.87,2.08]	39.8		0.18
Total (95% CI)	145	268	33	134	2.22 [1.19,4.14]	100.0	71	0.03^*^

CI, confidence interval; PASI, Psoriasis Area and Severity Index; PASI75, 75% improvement in the PASI from baseline; PASI50, 50% improvement in the PASI from baseline; PASI90, 90% improvement in the PASI from baseline; PASI100, 100% improvement in the PASI from baseline; sPGA, static Physician’s Global Assessment of Disease; sPGA 0/1, the score of static Physician’s Global Assessment of Disease achieves 0 or 1; sPGA 0, the score of static Physician’s Global Assessment of Disease achieves 0; CDLQI, Children’s Dermatology Life Quality Index; CDLQI 0/1, Children’s Dermatology Life Quality Index achieves 0 or 1; MTX, methotrexate. ^*^p < 0.05, ^#^p < 0.0001.

##### PASI 50

Two included articles employed PASI 50 as the outcome indicator, and the response rate with biologics was better than that with placebo at the endpoint (**Table 2**, **Data Sheet 2**; *P*-value = 0.27, RR = 2.83, 95%[CI] [2.20, 3.63]).

##### PASI 90

Compared with placebo treatment, ustekinumab, ixekizumab, and etanercept afforded 10.64-, 14.61-, and 4.1-fold better outcomes (*P*-value < 0.01), respectively, and the results further showed that secukinumab may be better than etanercept (**Table 2**, **Data Sheet 2**; *P-*value < 0.01, RR = 1.56, 95%[CI] [1.14, 2.15]). In summary, the PASI 90 of the biologics group was 3.85-fold higher than that of the control group (**Table 2**, **Data Sheet 2**; *P-*value < 0.01, RR = 3.85, 95%[CI] [1.40, 10.58]).

##### PASI 100

The PASI 100 score after ixekizumab treatment was markedly greater (27.76-fold better) than that after placebo treatment (**Table 2**; **Data Sheet 2**; *P-*value < 0.01, RR = 27.76, 95%[CI] [3.94, 195.31]), and adalimumab was 5.29-fold better than MTX treatment (**Table 2**, **Data Sheet 2**; *P-*value < 0.10, RR = 5.29, 95%[CI] [0.71, 39.42]). The PASI 100 response rate with biologics was 5.89-fold higher than that in the control group (**Table 2**, **Data Sheet 2**; *P-*value < 0.01, RR = 5.89, 95%[CI] [0.84, 41.51]).

##### Static Physician’s Global Assessment (sPGA)

The sPGA score assesses the average thickness, erythema, and scaling of psoriatic lesions, with scores ranging from 0 (clear) to 4 (severe), with 0/1 indicating clear or almost clear. Two studies used sPGA 0 as the outcome indicator, and the results were significant, favoring treatment with ustekinumab and ixekizumab over placebo (**Table 2**; **Data Sheet 2**; *P-*value = 0.62, RR = 22.01, 95%[CI] [5.53, 87.60]). Additionally, four reports used sPGA 0/1 as the evaluation indicator, demonstrating better efficacy following treatment with biologics (**Table 2**, **Data Sheet 2**; *P-*value < 0.01, RR = 3.15, 95%[CI] [1.26, 7.86]).

##### Children’s Dermatology Life Quality Index (CDLQI)

Three studies used the CDLQI scores to evaluate the general curative effect as a complete response. The biologics (ustekinumab and ixekizumab) showed better performance against psoriatic pathogenesis in adolescent patients (**Table 2**, **Data Sheet 2**). Overall, the efficacy outcomes were better in the biologic group than in the control group.

#### Safety

##### AEs

We compared among the groups. Surprisingly, there was no significant difference in the frequency of overall adverse reactions between the biologic and control groups. (**Table 3**, **Data Sheet 3**; *P-*value = 0.53, RR = 1.05, 95%[CI] [0.92, 1.19]) and fewer AEs were observed in the ustekinumab group than in the placebo group (*P-*value = 0.37, RR = 0.84, 95%[CI] [0.58, 1.22]).

**Table 3:** Adverse events and serious adverse events reported for biological agents used in pediatric patients with psoriasis.

Subgroup	Experimental		Control		Risk ratio M-H (95% CI)	Study weight, %	I^2^, %	P-value
	Events	Total	Events	Total				
**ALL AEs**								
Ustekinumab vs. Placebo	35	73	21	37	0.84 [0.58,1.22]	15.8		0.37
Ixekizumab vs. Placebo	64	115	25	56	1.25 [0.89,1.74]	19.1		0.20
Etanercept vs. Placebo	914	106	144	105	1.44 [0.81,1.60]	18.0		0.44
Secukinumab vs. Etanercept	68	80	34	41	1.02 [0.87,1.21]	25.5		0.77
Adalimumab vs. MTX	56	77	28	37	0.96 [0.76,1.21]	21.5		0.73
Total (95% CI)	1173	519	284	345	1.05 [0.92,1.19]	100.0	0	0.53
**Serious AEs**								
Ustekinumab vs. Placebo	1	73	0	37	1.54 [0.06,36.92]	4.8		0.79
Ixekizumab vs. Placebo	1	115	0	56	1.47 [0.06,35.62]	4.9		0.81
Etanercept vs. Placebo	4	106	3	105	1.32 [0.30,5.76]	22.1		0.71
Secukinumab vs. Etanercept	7	80	5	41	0.72 [0.24,2.12]	48.4		0.55
Adalimumab vs. MTX Total (95% CI)	9 22	77 451	2 10	37 276	2.16 [0.49,9.51] 1.21 [0.60,2.44]	19.8 100.0	0	0.31 0.82

CI, confidence interval; MTX, methotrexate; AEs, Adverse Events. ^*^p < 0.05, ^#^p < 0.000.

##### Serious AEs

We also compared the frequency of **serious adverse reactions** between the biologics and placebo and MTX groups. The probability of severe AEs was higher in the biologic group than in the control group (**Table 3**, **Data Sheet 4**; *P-*value = 0.82, RR = 1.21, 95%[CI] [0.60, 2.44]).

#### Specific adverse reactions

Type α AEs occur after the abundant release of inflammatory factors; these AEs are most frequent with complicated and changeable symptoms. Type β AEs are immune-mediated and are more serious than type α AEs, including immediate and delayed hypersensitivity reactions. Types γ and δ AEs are short- and long-term toxicities, respectively. Type ϵ AEs occur during drug withdrawal, particularly when a drug is suddenly stopped (14).

##### Type α

**Figure 3:**
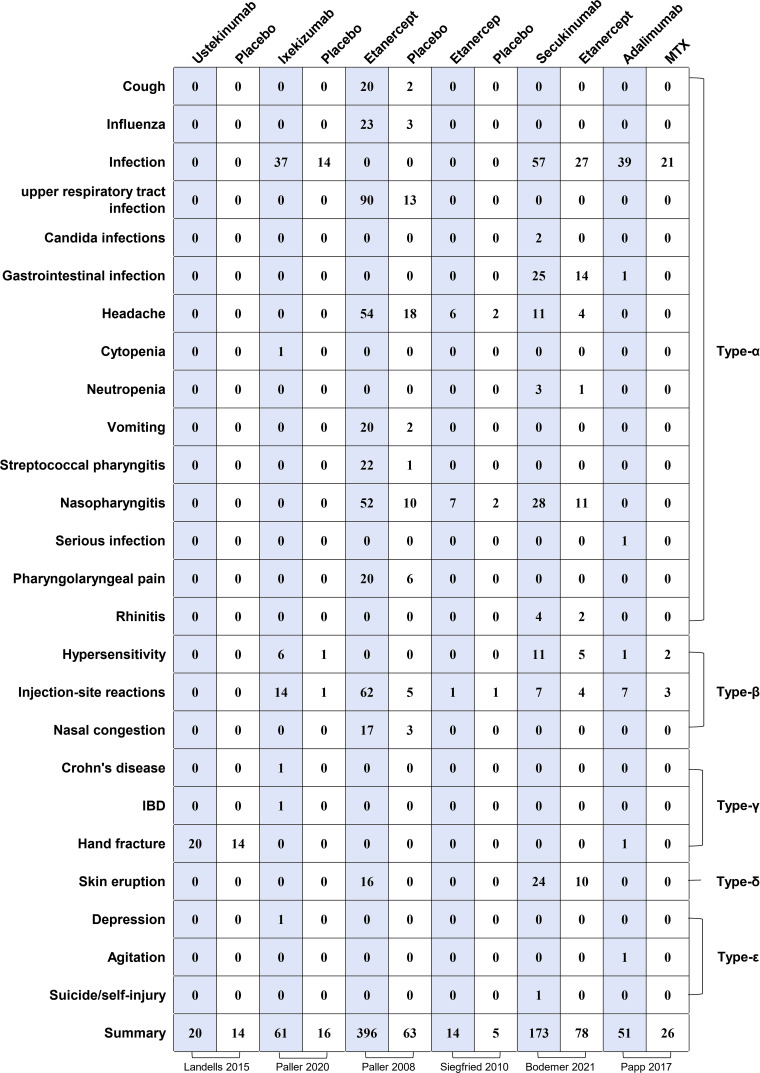
Mapping of specific adverse events between biological agents and control groups. MTX: methotrexate. IBD: inflammatory bowel disease.

In this study, type α AEs were the most frequently observed adverse reactions. The incidence of infection was 1.06-fold higher in the biologics group than in the control group (*P-*value = 0.48; RR = 1.06; 95%[CI] [0.87, 1.31]) (**Table 4**, **Data Sheet 5**). The occurrence of gastrointestinal infections was 0.93-fold lower in the biologic group than in the control group (*P-*value = 0.77; RR = 0.93; 95%[CI] [0.55, 1.59]). Furthermore, the probability of headaches was 2.65-fold greater in the biologic group than in the control group (*P-*value = 0.45; RR = 2.65; 95%[CI] [1.77, 3.98]). Serious infection in the biological group was 3.81-fold higher than that in the placebo treatment group (*P-*value = 0.48; RR = 3.81; 95%[CI] [0.49, 29.51]), for which no serious infection was reported. Other frequent AEs included cough, influenza, nausea, upper respiratory tract infection, Candida infection, cytopenia, neutropenia, vomiting, streptococcal pharyngitis, pharyngolaryngeal pain, and rhinitis (**Figure 3**).

**Table 4:** Specific adverse events reported on using biological agents in pediatric patients with psoriasis.

Subgroup	Experimental		Control		Risk ratio M-H(95% CI)	Study weight, %	I^2^, %	P-value
	Events	Total	Events	Total				
**Infection**								
Etanercept vs. Placebo	37	115	14	56	1.29 [0.76,2.18]	22.7		0.35
Secukinumab vs. Etanercept	57	80	27	41	1.08 [0.83,1.40]	43.1		0.55
Adalimumab vs. MTX	39	77	21	37	0.89 [0.62,1.28]	34.2		0.53
Total (95% CI)	133	272	62	134	1.06 [0.87,1.31]	100.0	0	0.48
**Gastrointestinal infection**								
Secukinumab vs. Etanercept	25	80	14	41	0.92 [0.54,1.56]	96.5		0.75
Adalimumab vs. MTX	1	77	0	37	1.46 [0.06,35.04]	3.5		0.81
Total (95% CI)	26	157	14	78	0.93 [0.55,1.59]	100.0	0	0.77
**Headache**								
Etanercept vs. Placebo	60	174	20	174	2.98 [1.91,4.64]	79.1		0.98
Secukinumab vs. Etanercept	11	80	4	41	1.41 [0.48,4.15]	20.9		0.53
Total (95% CI)	71	254	24	215	2.65 [1.77,3.98]	100.0	0	0.45
**Serious Infections**								
Ixekizumab vs. Placebo	1	115	0	56	1.47 [0.06,35.62]	57.2		0.81
Etanercept vs. Placebo	3	106	0	105	6.93 [0.36,132.62]	42.8		0.20
Total (95% CI)	4	221	0	161	3.81 [0.49,29.51]	100.0	0	0.48
**Hypersensitivity**								
Ixekizumab vs. Placebo	6	115	1	56	2.92 [0.36,23.69]	12.6		0.32
Secukinumab vs. Etanercept	12	80	5	41	1.23 [0.46,3.25]	62.0		0.68
Adalimumab vs. MTX	1	77	2	37	0.24 [0.02,2.57]	25.3		0.24
Total (95% CI)	19	272	8	134	1.19 [0.54,2.26]	100.0	19	0.29
**Injection-site reactions**								
Ixekizumab vs. Placebo	14	115	1	56	6.82 [0.92,50.55]	17.3		0.06
Etanercept vs. Placebo	63	174	6	174	5.02 [0.48,52.83]	37.8	65	0.18
Secukinumab vs. Etanercept	7	80	4	41	0.90 [0.28,2.89]	22.9		0.86
Adalimumab vs. MTX	7	77	3	37	1.12 [0.31,4.09]	22.1		0.86
Total (95% CI)	91	446	14	308	2.60 [0.66,10.25]	100.0	79	0.01
**Nasopharyngitis**								
Etanercept vs. Placebo	59	174	12	174	5.00 [2.89,8.67]	64.1		0.62
Secukinumab vs. Etanercept	28	80	11	41	1.30 [0.73,2.35]	38.6		0.37
Total (95% CI)	87	254	23	215	2.80 [0.95,8.19]	100.0	82	0.01^#^
**Hand fracture**								
Ustekinumab vs. Placebo	20	73	14	37	0.72 [0.41,1.26]	96.5		0.26
Adalimumab vs. MTX	1	77	0	37	1.46 [0.06,35.04]	3.5		0.81
Total (95% CI)	21	150	14	74	0.75 [0.43,1.30]	100.0	0	0.67
**Skin eruption**								
Etanercept vs. Placebo	16	106	0	105	32.69 [1.99,537.95]	44.0		0.01^#^
Secukinumab vs. Etanercept	24	80	10	41	1.23 [0.65,2.32]	56.0		0.52
Total (95% CI)	40	186	10	146	5.21 [0.10,260.11]	100.0	87	0.01^#^

CI, confidence interval; MTX, methotrexate; AEs, adverse events;^*^p < 0.05, ^#^p < 0.00 01.

##### Type β

In the present study, injection-site reactions were the most common type of β-adverse reactions, and the incidence of infection at the injection site was 2.60-fold higher in the biologics group than in the control group (*P-*value = 0.01; RR = 2.60; 95%[CI] [0.66, 10.25]) (**Table 4**, **Data Sheet 5**). Furthermore, the biologics group presented a 1.19-fold higher probability of hypersensitivity reactions than the control group (*P-*value = 0.29; RR = 1.19; 95%[CI] [0.54, 2.62]). Nasal congestion is another commonly reported type of β AE (**Figure 3**).

##### Type γ

The most frequently reported type γ adverse reaction was a hand fracture. The probability of hand fracture was 0.75-fold higher in the biologic group than in the control group (*P-*value = 0.67; RR = 0.75; 95%[CI] [0.43, 1.30]) (**Table 4**, **Data Sheet 5**). Other common type γ AEs are Crohn’s disease (CD) and irritable bowel disease; only one case of these two reactions, induced following ixekizumab therapy, was reported in the present study (**Figure 3**).

##### Type δ

Skin eruption was the only type δ adverse reaction reported, for which the biologic group presented a 5.21-fold higher probability than the control group (*P-*value = 0.006; RR = 5.21; 95%[CI] [0.10, 260.11]) (**Table 4**, **Data Sheet 5**) (**Figure 3**).

##### Type ϵ

Three types of adverse reactions, namely, depression, agitation, and self-injury, were documented in the biologics group (**Figure 3**).

### Bayesian analysis outcomes

**Figure 4:**
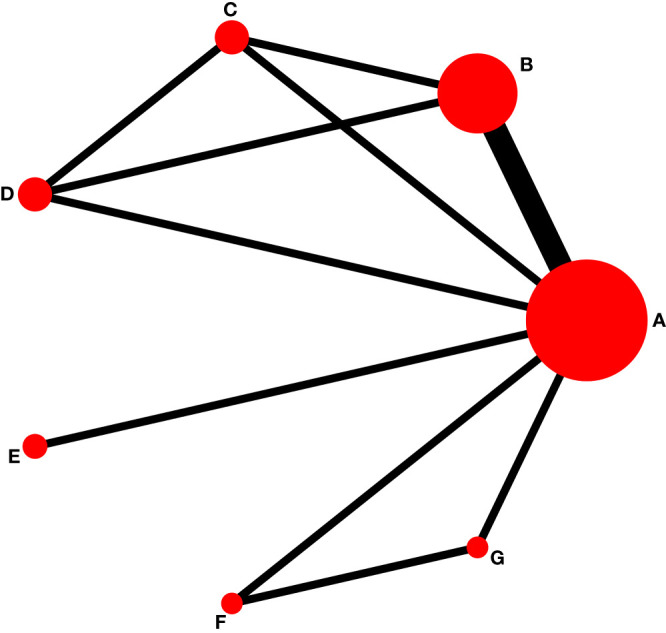
Network data on biologic agents in the treatment of psoriasis in children. LD, Low dose; HD, High dose. **(A)** placebo; **(B)** TNF-α Etanercept; **(C)** IL-17A Secukinumab LD; **(D)** IL-17A Secukinumab HD; **(E)** IL-17A Ixekizumab; **(F)** IL-23 Ustekinumab LD; **(G)** IL-23 Ustekinumab HD.

**Figure 5:**
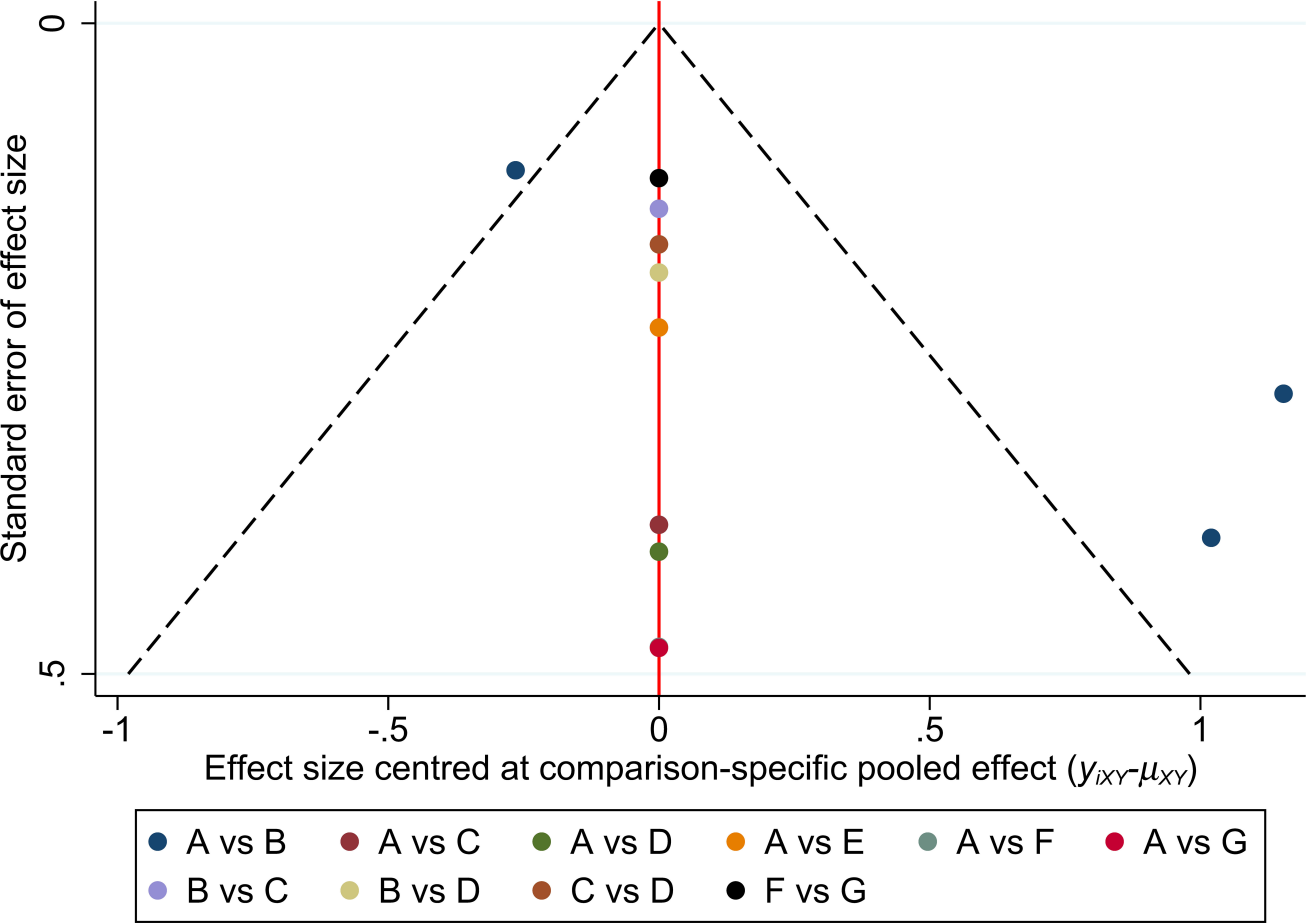
Comparison-adjusted funnel plot. A: placebo; B: TNF-α Etanercept; C: IL-17A Secukinumab LD; D: IL-17A Secukinumab HD; E: IL-17A Ixekizumab; F: IL-23 Ustekinumab LD; G: IL-23 Ustekinumab HD.

Five RCTs involving 791 patients reported a PASI of 75. The Bayesian analysis performed was a network meta-analysis (**Figure 4**). The size of the nodes represented the sample size of each study; the link between two nodes indicated direct comparisons, with the thickness of the lines representing the number of reports. No inconsistencies were detected when using the node-splitting method (*P-*value > 0.05) (**Data Sheet 6**). The scatter chart revealed a low bias (**Figure 5**).

**Figure 6:**
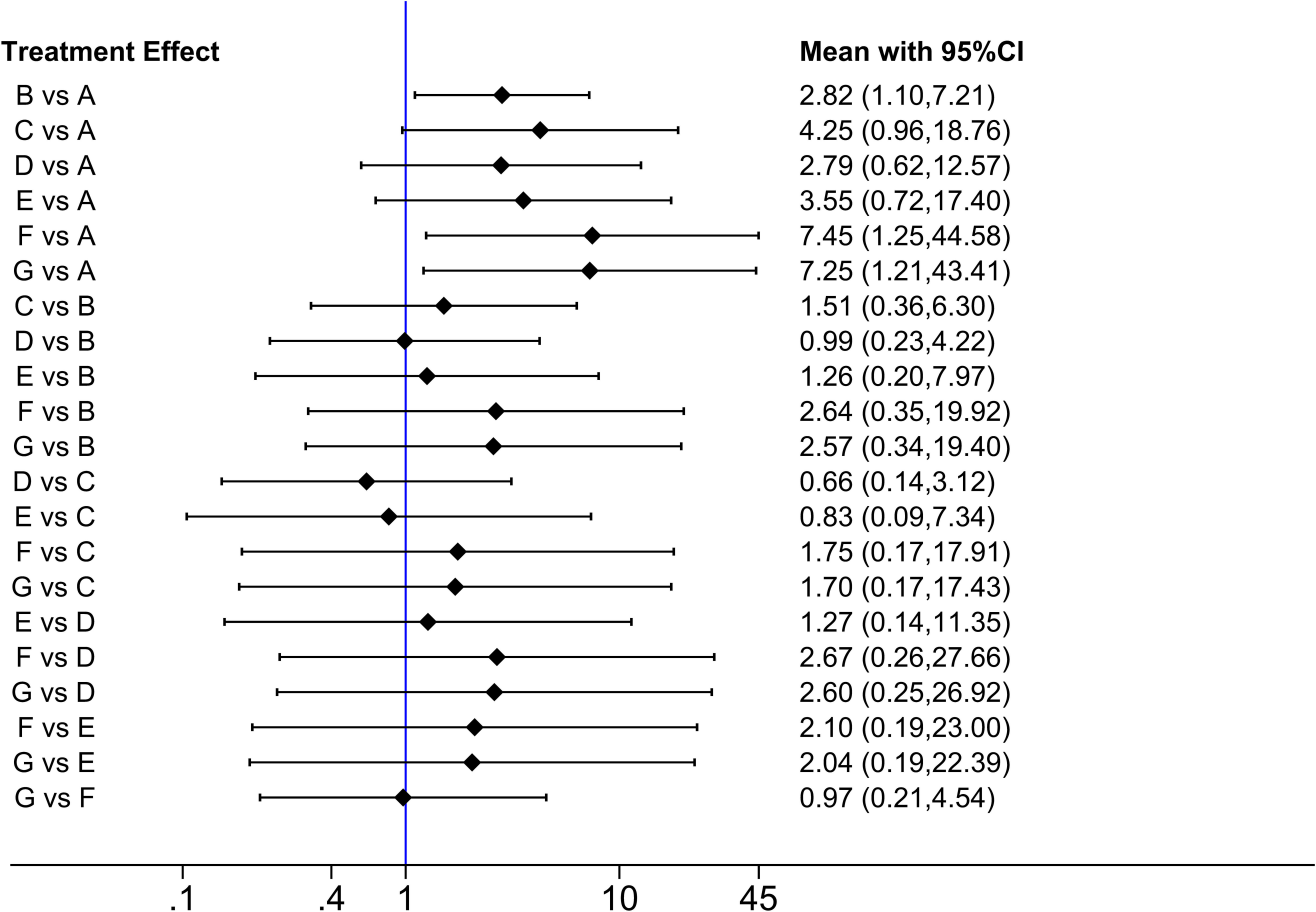
Meta-analysis forest plot. A: placebo; B: TNF-α Etanercept; C: IL-17A Secukinumab LD; D: IL-17A Secukinumab HD; E: IL-17A Ixekizumab; F: IL-23 Ustekinumab LD; G: IL-23 Ustekinumab HD.

Based on the Bayesian framework, we performed a meta-analysis using an MCMC random-effects model and generated 21 pairs of comparisons, of which three showed significant differences (**Figure 6**). Compared with that of A (placebo), the efficacies of B (TNF-α etanercept) (RR: 2.82, 95%[CI] [1.10,7.21]), F (IL-23 ustekinumab LD) (RR: 7.45, 95%[CI] [1.25,44.58]), and G (IL-23 ustekinumab HD) (RR: 7.25, 95%[CI] [1.21,43.41]) were superior. There were no other statistically significant differences.

**Figure 7:**
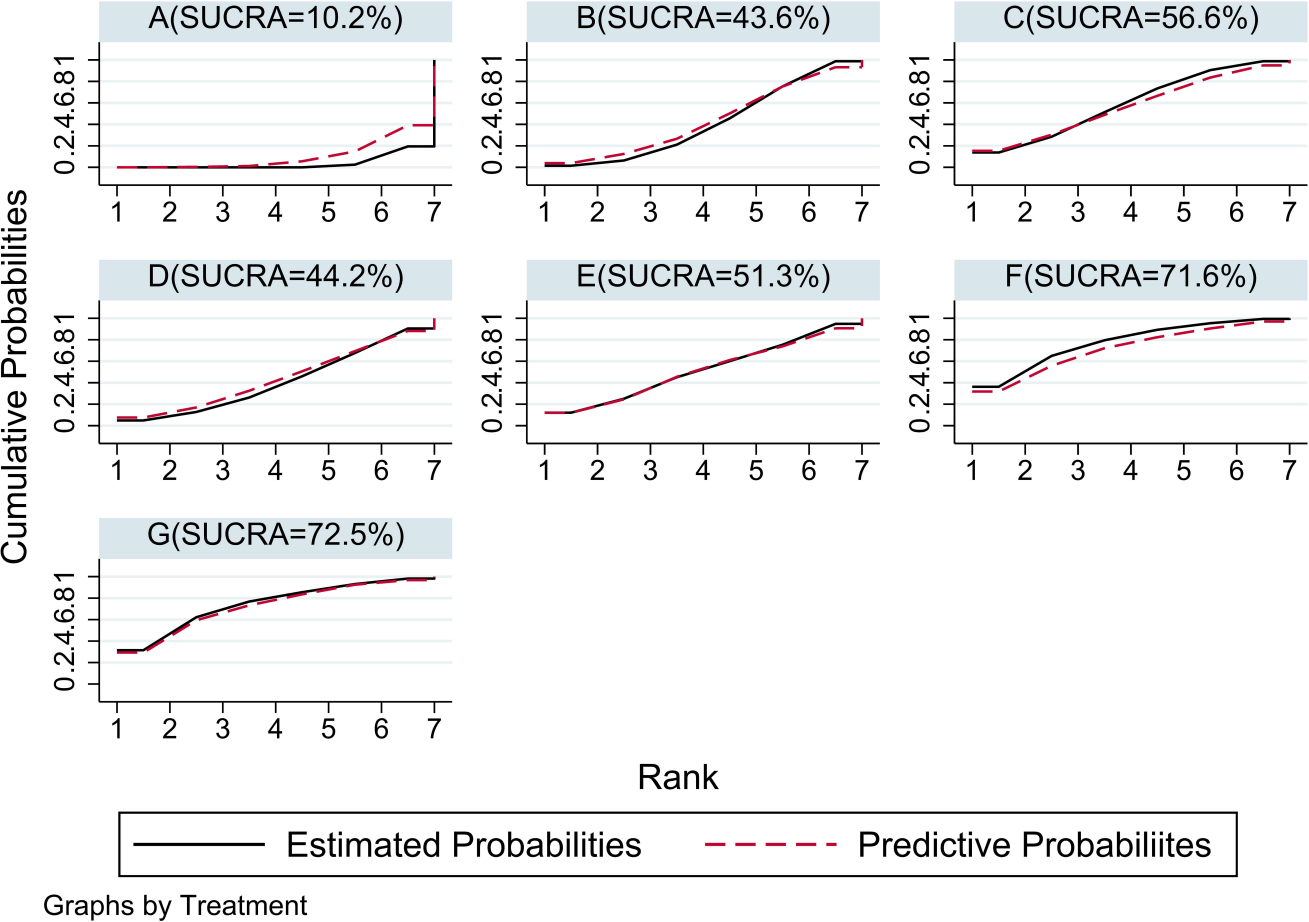
SUCRA rank plot of seven interventions. If the SUCRA approaches 100%, the intervention is the best among the included trials; conversely, if it is close to 0%, it represents the worst intervention. **(A)** placebo; **(B)** TNF-α Etanercept; **(C)** IL-17A Secukinumab LD; **(D)** IL-17A Secukinumab HD; **(E)** IL-17A Ixekizumab; **(F)** IL-23 Ustekinumab LD; **(G)** IL-23 Ustekinumab HD.

For the use of biologics in pediatric psoriasis patients, in this analysis, IL-23 ustekinumab HD(G) had the best efficacy (SUCRA = 72.5%), followed by IL-23 ustekinumab LD(F) (SUCRA = 71.6%)IL-17A Secukinumab (C) (SUCRA = 56.6%), IL-17A Ixekizumab (E) (SUCRA = 51.3%), IL-17A Secukinumab HD (D) (SUCRA = 44.2%), TNF-α Etanercept (B) (SUCRA = 43.6%), and placebo (A) (SUCRA = 10.2%). The SUCRA grade chart is shown in **Figure 7**. The efficacy comparison estimated using the ladder diagram is presented in **Table 5**. Bolded font indicates a statistical difference.

**Table 5:** Efficacy comparison of 7 interventions (random-effects model of MCMC method).

G	1.03 (0.22,4.80)	0.49 (0.04,5.36)	0.38 (0.04,3.99)	0.59 (0.06,6.00)	0.39 (0.05,2.94)	**0.14 (0.02,0.83)**
0.97 (0.21,4.54)	F	0.48 (0.04,5.21)	0.37 (0.04,3.88)	0.57 (0.06,5.83)	0.38 (0.05,2.85)	**0.13 (0.02,0.80)**
2.04 (0.19,22.39)	2.10 (0.19,23.00)	E	0.79 (0.09,7.03)	1.20 (0.14,10.55)	0.79 (0.13,5.04)	0.28 (0.06,1.38)
2.60 (0.25,26.92)	2.67 (0.26,27.66)	1.27 (0.14,11.35)	D	1.52 (0.32,7.24)	1.01 (0.24,4.31)	0.36 (0.08,1.61)
1.70 (0.17,17.43)	1.75 (0.17,17.91)	0.83 (0.09,7.34)	0.66 (0.14,3.12)	C	0.66 (0.16,2.77)	0.24 (0.05,1.04)
2.57 (0.34,19.40)	2.64 (0.35,19.92)	1.26 (0.20,7.97)	0.99 (0.23,4.22)	1.51 (0.36,6.30)	B	**0.35 (0.14,0.91)**
**7.25 (1.21,43.41)**	**7.45 (1.25,44.58)**	3.55 (0.72,17.40)	2.79 (0.62,12.57)	4.25 (0.96,18.76)	**2.82 (1.10,7.21)**	A

A: placebo; B: TNF-α Etanercept; C, IL-17A Secukinumab LD; D, IL-17A Secukinumab HD; E, IL-17A Ixekizumab; F, IL-23 Ustekinumab LD; G, IL-23 Ustekinumab HD.Bolded font indicates a statistical difference.

## Discussion

Juvenile-onset psoriasis is typically associated with a high probability of a positive family history and more serious symptoms, necessitating prompt therapeutic intervention (21). Treatment with biologics is the most effective for psoriasis, with a low incidence of adverse reactions in adults and a certain degree of safety (22). However, caution should be exercised when treating special populations, such as children. The present analysis provides comprehensive data on the use of biologics in children and adolescents with psoriasis. This is the first report of AEs in pediatric patients with psoriasis who were systematically exposed to biologics.

Studies have reported that the curative effect of biologics is considerable in pediatric patients compared to that in controls, and the AEs rates are similar to those in adults. In the present study, accumulated data suggested that anti-TNF-α preparations, such as etanercept, have lower efficacy, while adalimumab seemed more effective in treating psoriasis in children and adolescents. A previous study confirmed that the efficacy of etanercept may be similar to that of MTX (23, 24), which is consistent with our findings. Our Bayesian analysis showed differences in the efficacy of etanercept TNF-α inhibitor and ustekinumab IL-23 inhibitor compared with placebo. The highest dose of ustekinumab showed the best efficacy. Based on subjective evaluation scales (sPGA and CDLQI), ixekizumab was preferred for symptom improvement by clinicians, whereas ustekinumab was preferred by patients. The frequency of AEs between the biologics group and controls was almost equivalent, whereas the symptom severity was 1.22-fold higher in the biologics group than in the control group. These results indicated that the probability of AEs following treatment with biologics was not high, but rather more severe, which may decrease their acceptability.

To characterize the severe AEs caused by biologics, we classified and analyzed the different AEs induced by the biological agents. Accordingly, type α adverse reactions appeared to occur the most frequently. Children with psoriasis are prone to infections, headache, nasopharyngitis, injection-site infections, and skin eruptions. Compared to other biologics, etanercept is more likely to cause adverse reactions. Ustekinumab caused fewer AEs in children with psoriasis and could be recommended as a therapeutic option. In summary, our analysis showed that ustekinumab conferred better clinical efficacy with a low incidence of AEs, and hence could be recommended.

Herein, we reviewed retrospective RCTs evaluating biologics to determine cautionary issues and treatment risks during therapy in adolescents. This information could potentially enhance the quality and efficiency of future clinical research. The strengths of this study are as follows: (i) the included studies were of high quality, minimizing selection bias; (ii) all included studies had corresponding control groups, monitoring the bias and generating compelling evidence; and (iii) no pharmaceutical industry was involved in the present work. However, the analysis was limited by the small sample size, which was insufficient to determine the recommended dose and treatment duration of novel biological agents for pediatric patients. Future studies should focus on applying biologics in special populations, and clinicians should encourage relevant patients to enroll in prospective pharmacovigilance registries, undoubtedly helping to tackle unresolved questions in this field.

Finally, because of the short duration of use of biologics in children and the small number of relevant RCTs, the sample size of this study was not large enough, and the database search only yielded studies on secukinumab, ixekizumab, adalimumab, ustekinumab, and etanercept; the other biologics were not evaluated. Furthermore, the treatment duration of the included studies was not uniform and the recurrence rate was not reported.

## Conclusion

In conclusion, biologics can effectively treat children with psoriasis and can greatly improve their quality of life, eliminate lesions, and improve pruritus severity. Although AEs were reported in all the included studies, biologics can still be safely used to treat pediatric patients with moderate-to-severe psoriasis. Additionally, our analysis revealed that ustekinumab conferred good clinical efficacy with a low incidence of AEs, and hence could be recommended.

## Data availability statement

The original contributions presented in the study are included in the article/**Supplementary Material**. Further inquiries can be directed to the corresponding authors.

## Author contributions

XC and YR conceived the study. XC and YR designed this study. Y-qZ, YL, and JC performed literature search and data extraction. XC and MZ assessed the quality of the trials and analyzed the data. YR and CW drafted the original manuscript, and BL and XL contributed to the manuscript revision. All authors have read and approved the final manuscript.

## Funding

This work was supported by the National Natural Science Foundation of China (grant nos. 81874470 and 82074427), National Key Research and Development Program of China (grant no. 2018YFC1705302), Xinglin Scholar, Shanghai University of Traditional Chinese Medicine (No. RY411.14.12), Shanghai Pujiang Talent Program (No. 2020PJD067), Science and Technology Commission of Shanghai Municipality (Nos. 21Y21920100, 21Y21920102), and Shanghai Municipal Commission of Economy and Information Technology (No. 2020-RGZN-02038).

## Conflict of interest

The authors declare that the research was conducted in the absence of any commercial or financial relationships that could be construed as a potential conflict of interest.

## Publisher’s note

All claims expressed in this article are solely those of the authors and do not necessarily represent those of their affiliated organizations, or those of the publisher, the editors and the reviewers. Any product that may be evaluated in this article, or claim that may be made by its manufacturer, is not guaranteed or endorsed by the publisher.

## Abbreviations

T_H_1, T helper type 1 cells; T_H_17, T helper type 17 cells; TNF-α, tumor necrosis factor-α; IL-23, interleukin-23; IL-17, interleukin-17; IL-12, interleukin-12; PRISMA, Preferred Reporting Items for Systematic Review and Meta-Analysis; RCTs, randomized control trials; AEs, adverse events; PASI 75, Psoriasis Area and Severity Index 75; PASI 50/75/90/100, PASI score decreases more than 50%/75%/90%/100% from baseline; sPGA, Static Physician’s Global Assessment; sPGA0/1, Static Physician’s Global Assessment achieve 0 or 1; sPGA0, Static Physician’s Global Assessment achieved 0; CDQLI, Children’s Dermatology Life Quality Index; DLQI 0/1, Children’s Dermatology Life Quality Index achieve 0 or 1; RR, Risk ratios; CI, confidence intervals; MD, mean difference; SMD, standard mean difference; MTX, methotrexate; LD, Low dose; HD, High dose.
