# Supplementary material for: Efficacy and safety of biological agents for the treatment of pediatric patients with psoriasis: A bayesian analysis of six high-quality randomized controlled trials
Source: Front Immunol. 2022 Aug 19;13:896550. doi: 10.3389/fimmu.2022.896550 (PMC9446895; doi:10.3389/fimmu.2022.896550)
Supplement: Supplementary file 2 [file DataSheet_2.docx]

# PASI 75

**Experimental Control Risk Ratio**

**Risk Ratio**

**Risk of Bias**

**Study or Subgroup**

**Events**

**Total**

**Events**

**Total**

**Weight**

**M-H, Random, 95% CI**

**M-H, Random, 95% CI**

**A B C D E F**

- - 1. **Ustekinumab vs. Placebo**

| Landells 2015 | 58 | 73 | 4 | 37 | 13.6% | 7.35 [2.89, 18.68] | **+** | **+** | **+** | **+** | **+** | **+** |
| --- | --- | --- | --- | --- | --- | --- | --- | --- | --- | --- | --- | --- |
| **Subtotal (95% CI)**  Total events | 58 | **73** | 4 | **37** | **13.6%** | **7.35 [2.89, 18.68]** |  |  |  |  |  |  |

Heterogeneity: Not applicable

Test for overall effect: Z = 4.19 (P < 0.0001)

# Ixekizuman vs. Placebo

| Paller 2020 | 102 | 115 | 14 | 56 | 17.0% | 3.55 [2.24, 5.61] | **?** | **?** | **+** | **+** | **+** | **+** |
| --- | --- | --- | --- | --- | --- | --- | --- | --- | --- | --- | --- | --- |
| **Subtotal (95% CI)**  Total events | 102 | **115** | 14 | **56** | **17.0%** | **3.55 [2.24, 5.61]** |  |  |  |  |  |  |

Heterogeneity: Not applicable

Test for overall effect: Z = 5.42 (P < 0.00001)

# Etanercept vs. Placebo

| Paller 2008 | 60 | 106 | 12 | 105 | 16.4% | 4.95 [2.83, 8.65] | **+** | **+** | **+** | **+** | **+** | **+** |
| --- | --- | --- | --- | --- | --- | --- | --- | --- | --- | --- | --- | --- |
| Siegfried 2010 | 52 | 68 | 55 | 69 | 18.3% | 0.96 [0.80, 1.15] | **?** | **?** | **+** | **+** | **+** | **+** |
| **Subtotal (95% CI)** |  | **174** |  | **174** | **34.6%** | **2.15 [0.26, 18.04]** |  |  |  |  |  |  |
| Total events | 112 |  | 67 |  |  |  |  |  |  |  |  |  |

Heterogeneity: Tau² = 2.31; Chi² = 52.75, df = 1 (P < 0.00001); I² = 98% Test for overall effect: Z = 0.71 (P = 0.48)

# Secukinumab vs.Etanercept

| Bodemer 2021 | 72 | 80 | 28 | 41 | 18.1% | 1.32 [1.06, 1.64] | **?** | **?** | **+** | **+** | **+** | **+** |
| --- | --- | --- | --- | --- | --- | --- | --- | --- | --- | --- | --- | --- |
| **Subtotal (95% CI)**  Total events | 72 | **80** | 28 | **41** | **18.1%** | **1.32 [1.06, 1.64]** |  |  |  |  |  |  |

Heterogeneity: Not applicable

Test for overall effect: Z = 2.45 (P = 0.01)

| **1.1.5 Adalimumab vs. MTX** |  | | | | | | | | | | | |
| --- | --- | --- | --- | --- | --- | --- | --- | --- | --- | --- | --- | --- |
| Papp 2017 | 39 | 77 | 12 | 37 | 16.7% | 1.56 [0.93, 2.61] | **+** | **+** | **+** | **+** | **+** | **+** |
| **Subtotal (95% CI)** |  | **77** |  | **37** | **16.7%** | **1.56 [0.93, 2.61]** |  |  |  |  |  |  |
| Total events | 39 |  | 12 |  |  |  |  |  |  |  |  |  |

Heterogeneity: Not applicable

Test for overall effect: Z = 1.70 (P = 0.09)

| **Total (95% CI)** |  | **519** |  | **345** | **100.0%** | **2.37 [1.22, 4.62]** |
| --- | --- | --- | --- | --- | --- | --- |
| Total events | 383 |  | 125 |  |  |  |

Heterogeneity: Tau² = 0.62; Chi² = 106.65, df = 5 (P < 0.00001); I² = 95% Test for overall effect: Z = 2.55 (P = 0.01)

Test for subgroup differences: Chi² = 24.60, df = 4 (P < 0.0001), I² = 83.7%

Risk of bias legend

1. Random sequence generation (selection bias)
2. Allocation concealment (selection bias)
3. Blinding of participants and personnel (performance bias)
4. Blinding of outcome assessment (detection bias)
5. Incomplete outcome data (attrition bias)
6. Selective reporting (reporting bias)
7. Other bias

0.02 0.1 1 10 50

Control Experimental

# PASI 50

**Experimental Control Risk Ratio**

**Risk Ratio**

**Risk of Bias**

**Study or Subgroup**

**Events**

**Total**

**Events**

**Total**

**Weight**

**M-H, Fixed, 95% CI**

**M-H, Fixed, 95% CI**

**A B C D E F G**

- - 1. **Etanercept vs. Placebo**

Paller 2008

# Subtotal (95% CI)

Total events

79 106

# 106

79

24 105

# 105

24

46.1%

# 46.1%

3.26 [2.26, 4.71]

# 3.26 [2.26, 4.71]

**+ + + + + + +**

Heterogeneity: Not applicable

Test for overall effect: Z = 6.28 (P < 0.00001)

# Ixekizuman vs. Placebo

Paller 2020

# Subtotal (95% CI)

Total events

106

106

115

# 115

21 56

# 56

21

53.9%

# 53.9%

2.46 [1.75, 3.46]

# 2.46 [1.75, 3.46]

**? ? + + + + +**

Heterogeneity: Not applicable

Test for overall effect: Z = 5.15 (P < 0.00001)

| **Total (95% CI)** |  | **221** |  | **161** | **100.0%** | **2.83 [2.20, 3.63]** |
| --- | --- | --- | --- | --- | --- | --- |
| Total events | 185 |  | 45 |  |  |  |

Heterogeneity: Chi² = 1.22, df = 1 (P = 0.27); I² = 18% Test for overall effect: Z = 8.13 (P < 0.00001)

Test for subgroup differences: Chi² = 1.21, df = 1 (P = 0.27), I² = 17.5%

Risk of bias legend

1. Random sequence generation (selection bias)
2. Allocation concealment (selection bias)
3. Blinding of participants and personnel (performance bias)
4. Blinding of outcome assessment (detection bias)
5. Incomplete outcome data (attrition bias)
6. Selective reporting (reporting bias)
7. Other bias

0.05 0.2 1 5 20

Control Experimental

# PASI 90

**Experimental Control Risk Ratio**

**Risk Ratio**

**Risk of Bias**

**Study or Subgroup**

**Events**

**Total**

**Events**

**Total**

**Weight**

**M-H, Random, 95% CI**

**M-H, Random, 95% CI**

**A B C D E F**

- - 1. **Ustekinumab vs. Placebo**

| Landells 2015 | 42 | 73 | 2 | 37 | 16.5% | 10.64 [2.73, 41.56] | **+** | **+** | **+** | **+** | **+** | **+** |
| --- | --- | --- | --- | --- | --- | --- | --- | --- | --- | --- | --- | --- |
| **Subtotal (95% CI)**  Total events | 42 | **73** | 2 | **37** | **16.5%** | **10.64 [2.73, 41.56]** |  |  |  |  |  |  |

Heterogeneity: Not applicable

Test for overall effect: Z = 3.40 (P = 0.0007)

# Ixekizuman vs. Placebo

| Paller 2020 | 90 | 115 | 3 | 56 | 18.4% | 14.61 [4.84, 44.11] | **?** | **?** | **+** | **+** | **+** | **+** |
| --- | --- | --- | --- | --- | --- | --- | --- | --- | --- | --- | --- | --- |
| **Subtotal (95% CI)**  Total events | 90 | **115** | 3 | **56** | **18.4%** | **14.61 [4.84, 44.11]** |  |  |  |  |  |  |

Heterogeneity: Not applicable

Test for overall effect: Z = 4.76 (P < 0.00001)

# Etanercept vs. Placebo

| Paller 2008 | 29 | 106 | 7 | 105 | 20.7% | 4.10 [1.88, 8.95] | **+** | **+** | **+** | **+** | **+** | **+** |
| --- | --- | --- | --- | --- | --- | --- | --- | --- | --- | --- | --- | --- |
| **Subtotal (95% CI)**  Total events | 29 | **106** | 7 | **105** | **20.7%** | **4.10 [1.88, 8.95]** |  |  |  |  |  |  |

Heterogeneity: Not applicable

Test for overall effect: Z = 3.55 (P = 0.0004)

# Secukinumab vs.Etanercept

| Bodemer 2021 | 64 | 80 | 21 | 41 | 23.1% | 1.56 [1.14, 2.15] | **?** | **?** | **+** | **+** | **+** | **+** |
| --- | --- | --- | --- | --- | --- | --- | --- | --- | --- | --- | --- | --- |
| **Subtotal (95% CI)**  Total events | 64 | **80** | 21 | **41** | **23.1%** | **1.56 [1.14, 2.15]** |  |  |  |  |  |  |

Heterogeneity: Not applicable

Test for overall effect: Z = 2.75 (P = 0.006)

| **1.3.5 Adalimumab vs. MTX** |  | | | | | | | | | | | |
| --- | --- | --- | --- | --- | --- | --- | --- | --- | --- | --- | --- | --- |
| Papp 2017 | 23 | 77 | 8 | 37 | 21.2% | 1.38 [0.68, 2.79] | **+** | **+** | **+** | **+** | **+** | **+** |
| **Subtotal (95% CI)** |  | **77** |  | **37** | **21.2%** | **1.38 [0.68, 2.79]** |  |  |  |  |  |  |
| Total events | 23 |  | 8 |  |  |  |  |  |  |  |  |  |

Heterogeneity: Not applicable

Test for overall effect: Z = 0.90 (P = 0.37)

| **Total (95% CI)** |  | **451** |  | **276** | **100.0%** | **3.85 [1.40, 10.58]** |
| --- | --- | --- | --- | --- | --- | --- |
| Total events | 248 |  | 41 |  |  |  |

Heterogeneity: Tau² = 1.12; Chi² = 37.79, df = 4 (P < 0.00001); I² = 89% Test for overall effect: Z = 2.62 (P = 0.009)

Test for subgroup differences: Chi² = 24.80, df = 4 (P < 0.0001), I² = 83.9%

Risk of bias legend

1. Random sequence generation (selection bias)
2. Allocation concealment (selection bias)
3. Blinding of participants and personnel (performance bias)
4. Blinding of outcome assessment (detection bias)
5. Incomplete outcome data (attrition bias)
6. Selective reporting (reporting bias)
7. Other bias

0.005 0.1 1 10 200

Control Experimental

# PASI 100

**Experimental Control Risk Ratio**

**Risk Ratio**

**Risk of Bias**

**Study or Subgroup**

**Events**

**Total**

**Events**

**Total**

**Weight**

**M-H, Random, 95% CI**

**M-H, Random, 95% CI**

**A B C D E F**

- - 1. **Ixekizuman vs. Placebo**

| Paller 2020 | 57 | 115 | 1 | 56 | 29.9% | 27.76 [3.94, 195.31] | **?** | **?** | **+** | **+** | **+** | **+** |
| --- | --- | --- | --- | --- | --- | --- | --- | --- | --- | --- | --- | --- |
| **Subtotal (95% CI)**  Total events | 57 | **115** | 1 | **56** | **29.9%** | **27.76 [3.94, 195.31]** |  |  |  |  |  |  |

Heterogeneity: Not applicable

Test for overall effect: Z = 3.34 (P = 0.0008)

# Secukinumab vs.Etanercept

Bodemer 2021

# Subtotal (95% CI)

Total events

36 80

# 80

36

9 41

# 41

9

40.8%

# 40.8%

2.05 [1.10, 3.83]

# 2.05 [1.10, 3.83]

**? ? + + + +**

Heterogeneity: Not applicable

Test for overall effect: Z = 2.25 (P = 0.02)

| **1.4.3 Adalimumab vs. MTX** |  | | | | | | | | | | | |
| --- | --- | --- | --- | --- | --- | --- | --- | --- | --- | --- | --- | --- |
| Papp 2017 | 11 | 77 | 1 | 37 | 29.3% | 5.29 [0.71, 39.42] | **+** | **+** | **+** | **+** | **+** | **+** |
| **Subtotal (95% CI)** |  | **77** |  | **37** | **29.3%** | **5.29 [0.71, 39.42]** |  |  |  |  |  |  |
| Total events | 11 |  | 1 |  |  |  |  |  |  |  |  |  |

Heterogeneity: Not applicable

Test for overall effect: Z = 1.62 (P = 0.10)

| **Total (95% CI)** |  | **272** |  | **134** | **100.0%** | **5.89 [0.84, 41.51]** |
| --- | --- | --- | --- | --- | --- | --- |
| Total events | 104 |  | 11 |  |  |  |

Heterogeneity: Tau² = 2.33; Chi² = 9.99, df = 2 (P = 0.007); I² = 80% Test for overall effect: Z = 1.78 (P = 0.07)

Test for subgroup differences: Chi² = 6.65, df = 2 (P = 0.04), I² = 69.9%

Risk of bias legend

1. Random sequence generation (selection bias)
2. Allocation concealment (selection bias)
3. Blinding of participants and personnel (performance bias)
4. Blinding of outcome assessment (detection bias)
5. Incomplete outcome data (attrition bias)
6. Selective reporting (reporting bias)
7. Other bias

0.001 0.1 1 10 1000

Control Experimental

| **1.5 sPGA 0/1** | **Experimental** | | | **Control** | |  | **Risk Ratio** | **Risk Ratio** | **Risk of Bias** | | | | | | |
| --- | --- | --- | --- | --- | --- | --- | --- | --- | --- | --- | --- | --- | --- | --- | --- |
| **Study or Subgroup Events Total**  **1.5.1 Ustekinumab vs. Placebo** | | | | **Events Total** | | **Weight** | **M-H, Random, 95% CI** | **M-H, Random, 95% CI** | **A B C D E F** | | | | | | |
| Landells 2015 | 50 | | 73 | 2 | 37 | 15.2% | 12.67 [3.26, 49.22] | **+** | | **+** | **+** | **+** | **+** | **+** |  |
| **Subtotal (95% CI)** |  | | **73** |  | **37** | **15.2%** | **12.67 [3.26, 49.22]** |  | |  |  |  |  |  |  |
| Total events | 50 | |  | 2 |  |  |  |  | |  |  |  |  |  |  |
| Heterogeneity: Not applicable  Test for overall effect: Z = 3.67 (P = 0.0002)  **1.5.2 Ixekizuman vs. Placebo** | | | | | | | |  | |  | | | | |  |
| Paller 2020 | 93 | | 115 | 6 | 56 | 19.7% | 7.55 [3.53, 16.16] | **?** | | **?** | **+** | **+** | **+** | **+** |  |
| **Subtotal (95% CI)** |  | | **115** |  | **56** | **19.7%** | **7.55 [3.53, 16.16]** |  | |  |  |  |  |  |  |
| Total events | 93 | |  | 6 |  |  |  |  | |  |  |  |  |  |  |
| Heterogeneity: Not applicable  Test for overall effect: Z = 5.20 (P < 0.00001)  **1.5.3 Etanercept vs. Placebo** | | | | | | | |  | |  | | | | |  |
| Paller 2008 | 56 | | 106 | 14 | 105 | 21.3% | 3.96 [2.36, 6.66] | **+** | | **+** | **+** | **+** | **+** | **+** |  |
| Siegfried 2010 | 38 | | 68 | 35 | 69 | 22.2% | 1.10 [0.80, 1.51] | **?** | | **?** | **+** | **+** | **+** | **+** |  |
| **Subtotal (95% CI)** |  | | **174** |  | **174** | **43.5%** | **2.06 [0.54, 7.88]** |  | |  |  |  |  |  |  |
| Total events | 94 | |  | 49 |  |  |  |  | |  |  |  |  |  |  |
| Heterogeneity: Tau² = 0.89; Chi² = 19.55, df = 1 (P < 0.00001); I² = 95% Test for overall effect: Z = 1.05 (P = 0.29) | | | | | | | |  | |  | | | | |  |
| **1.5.4 Adalimumab vs. MTX** | |  |  |  |  |  |  |  | |  |  |  |  |  |  |
| Papp 2017 | | 39 | 77 | 15 | 37 | 21.6% | 1.25 [0.80, 1.96] | **+** | | **+** | **+** | **+** | **+** | **+** |  |
| **Subtotal (95% CI)** | |  | **77** |  | **37** | **21.6%** | **1.25 [0.80, 1.96]** |  | |  |  |  |  |  |  |
| Total events | | 39 |  | 15 |  |  |  |  | |  |  |  |  |  |  |
| Heterogeneity: Not applicable  Test for overall effect: Z = 0.97 (P = 0.33) | | | | | | | |  | | | | | | |  |

Heterogeneity: Tau² = 0.96; Chi² = 56.22, df = 4 (P < 0.00001); I² = 93% Test for overall effect: Z = 2.46 (P = 0.01)

| **Total (95% CI)** |  | **439** |  | **304** | **100.0%** | **3.15 [1.26, 7.86]** |
| --- | --- | --- | --- | --- | --- | --- |
| Total events | 276 |  | 72 |  |  |  |

Test for subgroup differences: Chi² = 22.58, df = 3 (P < 0.0001), I² = 86.7%

Risk of bias legend

1. Random sequence generation (selection bias)
2. Allocation concealment (selection bias)
3. Blinding of participants and personnel (performance bias)
4. Blinding of outcome assessment (detection bias)
5. Incomplete outcome data (attrition bias)
6. Selective reporting (reporting bias)
7. Other bias

0.005 0.1 1 10 200

Control Experimental

# sPGA 0

**Experimental Control Risk Ratio**

**Risk Ratio**

**Risk of Bias**

**Study or Subgroup**

**Events**

**Total**

**Events**

**Total**

**Weight**

**M-H, Fixed, 95% CI**

**M-H, Fixed, 95% CI**

**A B C D E F G**

- - 1. **Ustekinumab vs. Placebo**

| Landells 2015 | 29 | 73 | 1 | 37 | 49.7% 14.70 [2.08, 103.71] | **+ + + + + + +** |
| --- | --- | --- | --- | --- | --- | --- |
| **Subtotal (95% CI)**  Total events | 29 | **73** | 1 | **37** | **49.7% 14.70 [2.08, 103.71]** |  |

Heterogeneity: Not applicable

Test for overall effect: Z = 2.70 (P = 0.007)

# Ixekizuman vs. Placebo

| Paller 2020 | 60 | 115 | 1 | 56 | 50.3% 29.22 [4.16, 205.41] | **? ? + + + + +** |
| --- | --- | --- | --- | --- | --- | --- |
| **Subtotal (95% CI)**  Total events | 60 | **115** | 1 | **56** | **50.3% 29.22 [4.16, 205.41]** |  |

Heterogeneity: Not applicable

Test for overall effect: Z = 3.39 (P = 0.0007)

# Total (95% CI)

Total events

**188**

89

# 93 100.0%

2

# 22.01 [5.53, 87.60]

Heterogeneity: Chi² = 0.25, df = 1 (P = 0.62); I² = 0% Test for overall effect: Z = 4.39 (P < 0.0001)

Test for subgroup differences: Chi² = 0.24, df = 1 (P = 0.63), I² = 0%

Risk of bias legend

1. Random sequence generation (selection bias)
2. Allocation concealment (selection bias)
3. Blinding of participants and personnel (performance bias)
4. Blinding of outcome assessment (detection bias)
5. Incomplete outcome data (attrition bias)
6. Selective reporting (reporting bias)
7. Other bias

0.001 0.1 1 10 1000

Control Experimental

# CDLQI 0/1

**Experimental Control Risk Ratio**

**Risk Ratio**

**Risk of Bias**

**Study or Subgroup**

**Events**

**Total**

**Events**

**Total**

**Weight**

**M-H, Random, 95% CI**

**M-H, Random, 95% CI**

**A B C D E F**

- - 1. **Ustekinumab vs. Placebo**

Landells 2015

# Subtotal (95% CI)

Total events

29 73

# 73

29

4 37

# 37

4

22.6%

# 22.6%

3.67 [1.40, 9.67]

# 3.67 [1.40, 9.67]

**+ + + + + +**

Heterogeneity: Not applicable

Test for overall effect: Z = 2.64 (P = 0.008)

# Ixekizuman vs. Placebo

Paller 2020

# Subtotal (95% CI)

Total events

74 115

# 115

74

13 56

# 56

13

37.6%

# 37.6%

2.77 [1.69, 4.55]

# 2.77 [1.69, 4.55]

**? ? + + + +**

Heterogeneity: Not applicable

Test for overall effect: Z = 4.03 (P < 0.0001)

# Secukinumab vs.Etanercept

Bodemer 2021

# Subtotal (95% CI)

Total events

42 80

# 80

42

16 41

# 41

16

39.8%

# 39.8%

1.35 [0.87, 2.08]

# 1.35 [0.87, 2.08]

**? ? + + + +**

Heterogeneity: Not applicable

Test for overall effect: Z = 1.33 (P = 0.18)

| **Total (95% CI)** |  | **268** |  | **134** | **100.0%** | **2.22 [1.19, 4.14]** |
| --- | --- | --- | --- | --- | --- | --- |
| Total events | 145 |  | 33 |  |  |  |

Heterogeneity: Tau² = 0.21; Chi² = 6.79, df = 2 (P = 0.03); I² = 71% Test for overall effect: Z = 2.50 (P = 0.01)

Test for subgroup differences: Chi² = 6.36, df = 2 (P = 0.04), I² = 68.6%

Risk of bias legend

1. Random sequence generation (selection bias)
2. Allocation concealment (selection bias)
3. Blinding of participants and personnel (performance bias)
4. Blinding of outcome assessment (detection bias)
5. Incomplete outcome data (attrition bias)
6. Selective reporting (reporting bias)
7. Other bias

0.02 0.1 1 10 50

Control Experimental

# 2 AEs
