# Supplementary material for: Efficacy and safety of biological agents for the treatment of pediatric patients with psoriasis: A bayesian analysis of six high-quality randomized controlled trials
Source: Front Immunol. 2022 Aug 19;13:896550. doi: 10.3389/fimmu.2022.896550 (PMC9446895; doi:10.3389/fimmu.2022.896550)
Supplement: Supplementary file 3 [file DataSheet_3.docx]

biologics for psoriasis in children 01-Nov-2021

# ALL AEs

**Experimental Control Risk Ratio**

**Risk Ratio**

**Risk of Bias**

**Study or Subgroup**

**Events**

**Total**

**Events**

**Total**

**Weight**

**M-H, Fixed, 95% CI**

**M-H, Fixed, 95% CI**

**A B C D E F G**

- - 1. **Ustekinumab vs. Placebo**

| Landells 2015 | 35 | 73 | 21 | 37 | 15.8% | 0.84 [0.58, 1.22] | **+** | **+** | **+** | **+** | **+** | **+** | **+** |
| --- | --- | --- | --- | --- | --- | --- | --- | --- | --- | --- | --- | --- | --- |
| **Subtotal (95% CI)**  Total events | 35 | **73** | 21 | **37** | **15.8%** | **0.84 [0.58, 1.22]** |  |  |  |  |  |  |  |

Heterogeneity: Not applicable

Test for overall effect: Z = 0.90 (P = 0.37)

# Ixekizuman vs. Placebo

| Paller 2020 | 64 | 115 | 25 | 56 | 19.1% | 1.25 [0.89, 1.74] | **?** | **?** | **+** | **+** | **+** | **+** | **+** |
| --- | --- | --- | --- | --- | --- | --- | --- | --- | --- | --- | --- | --- | --- |
| **Subtotal (95% CI)**  Total events | 64 | **115** | 25 | **56** | **19.1%** | **1.25 [0.89, 1.74]** |  |  |  |  |  |  |  |

Heterogeneity: Not applicable

Test for overall effect: Z = 1.29 (P = 0.20)

# Etanercept vs. Placebo

| Paller 2008 | 914 | 106 | 144 | 105 |  | Not estimable | **+** | **+** | **+** | **+** | **+** | **+** | **+** |
| --- | --- | --- | --- | --- | --- | --- | --- | --- | --- | --- | --- | --- | --- |
| Siegfried 2010 | 36 | 68 | 32 | 69 | 18.0% | 1.14 [0.81, 1.60] | **?** | **?** | **+** | **+** | **+** | **+** | **+** |
| **Subtotal (95% CI)** |  | **174** |  | **174** | **18.0%** | **1.14 [0.81, 1.60]** |  |  |  |  |  |  |  |
| Total events | 950 |  | 176 |  |  |  |  |  |  |  |  |  |  |

Heterogeneity: Not applicable

Test for overall effect: Z = 0.77 (P = 0.44)

# Secukinumab vs.Etanercept

| Bodemer 2021 | 68 | 80 | 34 | 41 | 25.5% | 1.02 [0.87, 1.21] | **?** | **?** | **+** | **+** | **+** | **+** | **+** |
| --- | --- | --- | --- | --- | --- | --- | --- | --- | --- | --- | --- | --- | --- |
| **Subtotal (95% CI)**  Total events | 68 | **80** | 34 | **41** | **25.5%** | **1.02 [0.87, 1.21]** |  |  |  |  |  |  |  |

Heterogeneity: Not applicable

Test for overall effect: Z = 0.29 (P = 0.77)

| **2.1.5 Adalimumab vs. MTX** |  | | | | | | | | | | | | |
| --- | --- | --- | --- | --- | --- | --- | --- | --- | --- | --- | --- | --- | --- |
| Papp 2017 | 56 | 77 | 28 | 37 | 21.5% | 0.96 [0.76, 1.21] | **+** | **+** | **+** | **+** | **+** | **+** | **+** |
| **Subtotal (95% CI)**  Total events | 56 | **77** | 28 | **37** | **21.5%** | **0.96 [0.76, 1.21]** |  |  |  |  |  |  |  |

Heterogeneity: Not applicable

Test for overall effect: Z = 0.34 (P = 0.73)

| **Total (95% CI)** |  | **519** |  | **345** | **100.0%** | **1.05 [0.92, 1.19]** |
| --- | --- | --- | --- | --- | --- | --- |
| Total events | 1173 |  | 284 |  |  |  |

Heterogeneity: Chi² = 3.19, df = 4 (P = 0.53); I² = 0% Test for overall effect: Z = 0.71 (P = 0.48)

Test for subgroup differences: Chi² = 3.07, df = 4 (P = 0.55), I² = 0%

Risk of bias legend

1. Random sequence generation (selection bias)
2. Allocation concealment (selection bias)
3. Blinding of participants and personnel (performance bias)
4. Blinding of outcome assessment (detection bias)
5. Incomplete outcome data (attrition bias)
6. Selective reporting (reporting bias)
7. Other bias

0.2 0.5 1 2 5

Control Experimental

Review Manager 5.4.1 9
