# Supplementary material for: Efficacy and safety of biological agents for the treatment of pediatric patients with psoriasis: A bayesian analysis of six high-quality randomized controlled trials
Source: Front Immunol. 2022 Aug 19;13:896550. doi: 10.3389/fimmu.2022.896550 (PMC9446895; doi:10.3389/fimmu.2022.896550)
Supplement: Supplementary file 4 [file DataSheet_4.docx]

| biologics for psoriasis in children |  |  |  | 01-Nov-2021 |
| --- | --- | --- | --- | --- |
| **2.2 Serious AEs** |  |  |  |  |
| **Experimental Control Study or Subgroup Events Total Events Total** | **Weight** | **Risk Ratio**  **M-H, Fixed, 95% CI** | **Risk Ratio**  **M-H, Fixed, 95% CI** | **Risk of Bias**  **A B C D E F G** |

# Ustekinumab vs. Placebo

| Landells 2015 | 1 | 73 | 0 | 37 | 4.8% | 1.54 [0.06, 36.92] | **+** | **+** | **+** | **+** | **+** | **+** | **+** |
| --- | --- | --- | --- | --- | --- | --- | --- | --- | --- | --- | --- | --- | --- |
| **Subtotal (95% CI)** |  | **73** |  | **37** | **4.8%** | **1.54 [0.06, 36.92]** |  | | | | | | |
| Total events  Heterogeneity: Not applicable | 1 |  | 0 |  |  |  |  |  |  |  |  |  |  |

Test for overall effect: Z = 0.27 (P = 0.79)

# Ixekizuman vs. Placebo

| Paller 2020 | 1 | 115 | 0 | 56 | 4.9% | 1.47 [0.06, 35.62] | **?** | **?** | **+** | **+** | **+** | **+** | **+** |
| --- | --- | --- | --- | --- | --- | --- | --- | --- | --- | --- | --- | --- | --- |
| **Subtotal (95% CI)** |  | **115** |  | **56** | **4.9%** | **1.47 [0.06, 35.62]** |  |  |  |  |  |  |  |
| Total events | 1 |  | 0 |  |  |  |  |  |  |  |  |  |  |
| Heterogeneity: Not applicable |  |  |  |  |  |  |  |  |  |  |  |  |  |
| Test for overall effect: Z = 0.24 (P = 0.81) | | | | | | | | | | | | | |
| **2.2.3 Etanercept vs. Placebo**  Paller 2008 | 4 | 106 | 3 | 105 | 22.1% | 1.32 [0.30, 5.76] | **+** | **+** | **+** | **+** | **+** | **+** | **+** |
| **Subtotal (95% CI)** |  | **106** |  | **105** | **22.1%** | **1.32 [0.30, 5.76]** |  |  |  |  |  |  |  |
| Total events | 4 |  | 3 |  |  |  |  |  |  |  |  |  |  |
| Heterogeneity: Not applicable |  |  |  |  |  |  |  |  |  |  |  |  |  |

Test for overall effect: Z = 0.37 (P = 0.71)

# 2.2.4 Secukinumab vs.Etanercept

Bodemer 2021 7 80

# Subtotal (95% CI) 80

Total events 7

Heterogeneity: Not applicable

5 41

# 41

5

48.4%

# 48.4%

0.72 [0.24, 2.12]

# 0.72 [0.24, 2.12]

**? ? + + + + +**

Test for overall effect: Z = 0.60 (P = 0.55)

| **2.2.5 Adalimumab vs. MTX** |  | | | | | | | | | | | | |
| --- | --- | --- | --- | --- | --- | --- | --- | --- | --- | --- | --- | --- | --- |
| Papp 2017 | 9 | 77 | 2 | 37 | 19.8% | 2.16 [0.49, 9.51] | **+** | **+** | **+** | **+** | **+** | **+** | **+** |
| **Subtotal (95% CI)** |  | **77** |  | **37** | **19.8%** | **2.16 [0.49, 9.51]** |  |  |  |  |  |  |  |
| Total events  Heterogeneity: Not applicable | 9 |  | 2 |  |  |  |  |  |  |  |  |  |  |

Test for overall effect: Z = 1.02 (P = 0.31)

# Total (95% CI)

Total events

**451**

22

**276**

10

# 100.0%

**1.21 [0.60, 2.44]**

Heterogeneity: Chi² = 1.54, df = 4 (P = 0.82); I² = 0% Test for overall effect: Z = 0.54 (P = 0.59)

Test for subgroup differences: Chi² = 1.52, df = 4 (P = 0.82), I² = 0%

Risk of bias legend

1. Random sequence generation (selection bias)
2. Allocation concealment (selection bias)
3. Blinding of participants and personnel (performance bias)
4. Blinding of outcome assessment (detection bias)
5. Incomplete outcome data (attrition bias)
6. Selective reporting (reporting bias)
7. Other bias

0.001 0.1 1 10 1000

Control Experimental

# 3 Others AE

Review Manager 5.4.1 10
