# Supplementary material for: Efficacy and safety of biological agents for the treatment of pediatric patients with psoriasis: A bayesian analysis of six high-quality randomized controlled trials
Source: Front Immunol. 2022 Aug 19;13:896550. doi: 10.3389/fimmu.2022.896550 (PMC9446895; doi:10.3389/fimmu.2022.896550)
Supplement: Supplementary file 5 [file DataSheet_5.docx]

# Others AE

- 1. **Infection**

**Experimental Control Risk Ratio**

**Risk Ratio**

**Risk of Bias**

**Study or Subgroup**

**Events**

**Total**

**Events**

**Total**

**Weight**

**M-H, Fixed, 95% CI**

**M-H, Fixed, 95% CI**

**A B C D E F G**

- - 1. **Etanercept vs. Placebo**

Siegfried 2010

# Subtotal (95% CI)

Total events

37 115

# 115

37

14 56

# 56

14

22.7%

# 22.7%

1.29 [0.76, 2.18]

# 1.29 [0.76, 2.18]

**? ? + + + + +**

Heterogeneity: Not applicable

Test for overall effect: Z = 0.94 (P = 0.35)

# Secukinumab vs.Etanercept

Bodemer 2021

# Subtotal (95% CI)

Total events

57 80

# 80

57

27 41

# 41

27

43.1%

# 43.1%

1.08 [0.83, 1.40]

# 1.08 [0.83, 1.40]

**? ? + + + + +**

Heterogeneity: Not applicable

Test for overall effect: Z = 0.59 (P = 0.55)

| **3.1.3 Adalimumab vs. MTX** |  | | | | | | | | | | | | |
| --- | --- | --- | --- | --- | --- | --- | --- | --- | --- | --- | --- | --- | --- |
| Papp 2017 | 39 | 77 | 21 | 37 | 34.2% | 0.89 [0.62, 1.28] | **+** | **+** | **+** | **+** | **+** | **+** | **+** |
| **Subtotal (95% CI)**  Total events | 39 | **77** | 21 | **37** | **34.2%** | **0.89 [0.62, 1.28]** |  |  |  |  |  |  |  |

Heterogeneity: Not applicable

Test for overall effect: Z = 0.62 (P = 0.53)

# Total (95% CI)

Total events

133

**272**

**134**

62

# 100.0%

**1.06 [0.87, 1.31]**

Heterogeneity: Chi² = 1.45, df = 2 (P = 0.48); I² = 0% Test for overall effect: Z = 0.59 (P = 0.56)

Test for subgroup differences: Chi² = 1.42, df = 2 (P = 0.49), I² = 0%

Risk of bias legend

1. Random sequence generation (selection bias)
2. Allocation concealment (selection bias)
3. Blinding of participants and personnel (performance bias)
4. Blinding of outcome assessment (detection bias)
5. Incomplete outcome data (attrition bias)
6. Selective reporting (reporting bias)
7. Other bias

0.2 0.5 1 2 5

Control Experimental

# Gastrointestinal infection

|  | **Experimental** | | **Control** | |  | **Risk Ratio** | **Risk Ratio** | **Risk of Bias** |
| --- | --- | --- | --- | --- | --- | --- | --- | --- |
| **Study or Subgroup** | **Events Total** | | **Events Total** | | **Weight** | **M-H, Fixed, 95% CI** | **M-H, Fixed, 95% CI** | **A B C D E F G** |
| **3.2.1 Secukinumab vs.Etanercept** | | | | | | | | |
| Bodemer 2021 | 25 | 80 | 14 | 41 | 96.5% | 0.92 [0.54, 1.56] | **? ? + + + + +** | |
| **Subtotal (95% CI)** |  | **80** |  | **41** | **96.5%** | **0.92 [0.54, 1.56]** |  | |
| Total events | 25 |  | 14 |  |  |  |  | |

Heterogeneity: Not applicable

Test for overall effect: Z = 0.32 (P = 0.75)

| **3.2.2 Adalimumab vs. MTX** |  | | | | | | |
| --- | --- | --- | --- | --- | --- | --- | --- |
| Papp 2017 | 1 | 77 | 0 | 37 | 3.5% | 1.46 [0.06, 35.04] | **+ + + + + + +** |
| **Subtotal (95% CI)** |  | **77** |  | **37** | **3.5%** | **1.46 [0.06, 35.04]** |  |
| Total events  Heterogeneity: Not applicable | 1 |  | 0 |  |  |  |  |
| Test for overall effect: Z = 0.23 (P = 0.81) | | | | | | | |
| **Total (95% CI)** |  | **157** |  | **78** | **100.0%** | **0.93 [0.55, 1.59]** | |
| Total events | 26 |  | 14 |  |  |  | |

Heterogeneity: Chi² = 0.08, df = 1 (P = 0.77); I² = 0% Test for overall effect: Z = 0.25 (P = 0.80)

Test for subgroup differences: Chi² = 0.08, df = 1 (P = 0.78), I² = 0%

Risk of bias legend

1. Random sequence generation (selection bias)
2. Allocation concealment (selection bias)
3. Blinding of participants and personnel (performance bias)
4. Blinding of outcome assessment (detection bias)
5. Incomplete outcome data (attrition bias)
6. Selective reporting (reporting bias)
7. Other bias

0.001 0.1 1 10 1000

Control Experimental

# Headache

**Experimental Control Risk Ratio**

**Risk Ratio**

**Risk of Bias**

**Study or Subgroup**

**Events**

**Total**

**Events**

**Total**

**Weight**

**M-H, Fixed, 95% CI**

**M-H, Fixed, 95% CI**

**A B C D E F G**

- - 1. **Etanercept vs. Placebo**

| Paller 2008 | 54 | 106 | 18 | 105 | 71.3% | 2.97 [1.88, 4.71] | **+** | **+** | **+** | **+** | **+** | **+** | **+** |
| --- | --- | --- | --- | --- | --- | --- | --- | --- | --- | --- | --- | --- | --- |
| Siegfried 2010 | 6 | 68 | 2 | 69 | 7.8% | 3.04 [0.64, 14.56] | **?** | **?** | **+** | **+** | **+** | **+** | **+** |
| **Subtotal (95% CI)** |  | **174** |  | **174** | **79.1%** | **2.98 [1.91, 4.64]** |  |  |  |  |  |  |  |
| Total events | 60 |  | 20 |  |  |  |  |  |  |  |  |  |  |

Heterogeneity: Chi² = 0.00, df = 1 (P = 0.98); I² = 0% Test for overall effect: Z = 4.83 (P < 0.00001)

# Secukinumab vs.Etanercept

| Bodemer 2021 | 11 | 80 | 4 | 41 | 20.9% | 1.41 [0.48, 4.15] | **?** | **?** | **+** | **+** | **+** | **+** | **+** |
| --- | --- | --- | --- | --- | --- | --- | --- | --- | --- | --- | --- | --- | --- |
| **Subtotal (95% CI)**  Total events | 11 | **80** | 4 | **41** | **20.9%** | **1.41 [0.48, 4.15]** |  |  |  |  |  |  |  |

Heterogeneity: Not applicable

Test for overall effect: Z = 0.62 (P = 0.53)

# Total (95% CI)

Total events

**254**

71

**215**

24

# 100.0%

**2.65 [1.77, 3.98]**

Heterogeneity: Chi² = 1.58, df = 2 (P = 0.45); I² = 0% Test for overall effect: Z = 4.71 (P < 0.00001)

Test for subgroup differences: Chi² = 1.58, df = 1 (P = 0.21), I² = 36.6%

Risk of bias legend

1. Random sequence generation (selection bias)
2. Allocation concealment (selection bias)
3. Blinding of participants and personnel (performance bias)
4. Blinding of outcome assessment (detection bias)
5. Incomplete outcome data (attrition bias)
6. Selective reporting (reporting bias)
7. Other bias

0.001 0.1 1 10 1000

Control Experimental

| **3.4 Serious Infections** | **Experimental** | | | **Control** | |  | **Risk Ratio** | **Risk Ratio** | **Risk of Bias** |
| --- | --- | --- | --- | --- | --- | --- | --- | --- | --- |
| **Study or Subgroup** | **Events Total** | | | **Events Total** | | **Weight** | **M-H, Fixed, 95% CI** | **M-H, Fixed, 95% CI** | **A B C D E F G** |
| **3.4.1 Ixekizuman vs. Placebo** | | | | | | | | | |
| Paller 2020 | | 1 | 115 | 0 | 56 | 57.2% | 1.47 [0.06, 35.62] | **? ? + + + + +** | |
| **Subtotal (95% CI)** | |  | **115** |  | **56** | **57.2%** | **1.47 [0.06, 35.62]** |  | |
| Total events | | 1 |  | 0 |  |  |  |  | |
| Heterogeneity: Not applicable | |  |  |  |  |  |  |  | |
| Test for overall effect: Z = 0.24 (P = 0.81) | | | | | | | | | |
| **3.4.2 Etanercept vs. Placebo**  Paller 2008 | | 3 | 106 | 0 | 105 | 42.8% | 6.93 [0.36, 132.62] | **+ + + + + + +** | |
| **Subtotal (95% CI)** | |  | **106** |  | **105** | **42.8%** | **6.93 [0.36, 132.62]** |  | |
| Total events | | 3 |  | 0 |  |  |  |  | |
| Heterogeneity: Not applicable | |  |  |  |  |  |  |  | |
| Test for overall effect: Z = 1.29 (P = 0.20) | | | | | | | | | |

# Total (95% CI)

Total events

**221**

4

**161**

0

# 100.0%

**3.81 [0.49, 29.51]**

Heterogeneity: Chi² = 0.50, df = 1 (P = 0.48); I² = 0% Test for overall effect: Z = 1.28 (P = 0.20)

Test for subgroup differences: Chi² = 0.49, df = 1 (P = 0.48), I² = 0%

Risk of bias legend

1. Random sequence generation (selection bias)
2. Allocation concealment (selection bias)
3. Blinding of participants and personnel (performance bias)
4. Blinding of outcome assessment (detection bias)
5. Incomplete outcome data (attrition bias)
6. Selective reporting (reporting bias)
7. Other bias

0.001 0.1 1 10 1000

Control Experimental

# Hypersensitivty

**Experimental Control Risk Ratio**

**Risk Ratio**

**Risk of Bias**

**Study or Subgroup**


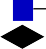


**Events**

**Total**

**Events**

**Total**

**Weight**

**M-H, Fixed, 95% CI**

**M-H, Fixed, 95% CI**

**A B C D E F G**

- - 1. **Ixekizuman vs. Placebo**

| Paller 2020 | 6 | 115 | 1 | 56 | 12.6% | 2.92 [0.36, 23.69] | **?** | **?** | **+** | **+** | **+** | **+** | **+** |
| --- | --- | --- | --- | --- | --- | --- | --- | --- | --- | --- | --- | --- | --- |
| **Subtotal (95% CI)** |  | **115** |  | **56** | **12.6%** | **2.92 [0.36, 23.69]** |  | | | | | | |
| Total events  Heterogeneity: Not applicable | 6 |  | 1 |  |  |  |  |  |  |  |  |  |  |

Test for overall effect: Z = 1.00 (P = 0.32)

# Secukinumab vs.Etanercept

Bodemer 2021

# Subtotal (95% CI)

Total events

12 80

# 80

12

5 41

# 41

5

62.0%

# 62.0%

1.23 [0.46, 3.25]

# 1.23 [0.46, 3.25]

**? ? + + + + +**

Heterogeneity: Not applicable

Test for overall effect: Z = 0.42 (P = 0.68)

| **3.5.3 Adalimumab vs. MTX** |  | | | | | | | | | | | | |
| --- | --- | --- | --- | --- | --- | --- | --- | --- | --- | --- | --- | --- | --- |
| Papp 2017 | 1 | 77 | 2 | 37 | 25.3% | 0.24 [0.02, 2.57] | **+** | **+** | **+** | **+** | **+** | **+** | **+** |
| **Subtotal (95% CI)** |  | **77** |  | **37** | **25.3%** | **0.24 [0.02, 2.57]** |  |  |  |  |  |  |  |
| Total events  Heterogeneity: Not applicable | 1 |  | 2 |  |  |  |  |  |  |  |  |  |  |

Test for overall effect: Z = 1.18 (P = 0.24)

# Total (95% CI)

Total events

**272**

19

**134**

8

# 100.0%

**1.19 [0.54, 2.62]**

Heterogeneity: Chi² = 2.47, df = 2 (P = 0.29); I² = 19% Test for overall effect: Z = 0.44 (P = 0.66)

Test for subgroup differences: Chi² = 2.46, df = 2 (P = 0.29), I² = 18.7%

Risk of bias legend

1. Random sequence generation (selection bias)
2. Allocation concealment (selection bias)
3. Blinding of participants and personnel (performance bias)
4. Blinding of outcome assessment (detection bias)
5. Incomplete outcome data (attrition bias)
6. Selective reporting (reporting bias)
7. Other bias

0.001 0.1 1 10 1000

Control Experimental

# Injection-site reactions

**Experimental Control Risk Ratio**

**Risk Ratio**

**Risk of Bias**

**Study or Subgroup**

**Events**

**Total**

**Events**

**Total**

**Weight**

**M-H, Random, 95% CI**

**M-H, Random, 95% CI**

**A B C D E F**

- - 1. **Ixekizuman vs. Placebo**

| Paller 2020 | 14 | 115 | 1 | 56 | 17.3% | 6.82 [0.92, 50.55] | **?** | **?** | **+** | **+** | **+** | **+** |
| --- | --- | --- | --- | --- | --- | --- | --- | --- | --- | --- | --- | --- |
| **Subtotal (95% CI)**  Total events | 14 | **115** | 1 | **56** | **17.3%** | **6.82 [0.92, 50.55]** |  |  |  |  |  |  |

Heterogeneity: Not applicable

Test for overall effect: Z = 1.88 (P = 0.06)

# Etanercept vs. Placebo

| Paller 2008 | 62 | 106 | 5 | 105 | 24.7% | 12.28 [5.14, 29.33] | **+** | **+** | **+** | **+** | **+** | **+** |
| --- | --- | --- | --- | --- | --- | --- | --- | --- | --- | --- | --- | --- |
| Siegfried 2010 | 1 | 68 | 1 | 69 | 13.1% | 1.01 [0.06, 15.90] | **?** | **?** | **+** | **+** | **+** | **+** |
| **Subtotal (95% CI)** |  | **174** |  | **174** | **37.8%** | **5.02 [0.48, 52.83]** |  |  |  |  |  |  |
| Total events | 63 |  | 6 |  |  |  |  |  |  |  |  |  |

Heterogeneity: Tau² = 2.05; Chi² = 2.89, df = 1 (P = 0.09); I² = 65% Test for overall effect: Z = 1.34 (P = 0.18)

# Secukinumab vs.Etanercept

| Bodemer 2021 | 7 | 80 | 4 | 41 | 22.9% | 0.90 [0.28, 2.89] | **?** | **?** | **+** | **+** | **+** | **+** |
| --- | --- | --- | --- | --- | --- | --- | --- | --- | --- | --- | --- | --- |
| **Subtotal (95% CI)** |  | **80** |  | **41** | **22.9%** | **0.90 [0.28, 2.89]** |  |  |  |  |  |  |
| Total events | 7 |  | 4 |  |  |  |  |  |  |  |  |  |
| Heterogeneity: Not applicable |  |  |  |  |  |  |  |  |  |  |  |  |
| Test for overall effect: Z = 0.18 (P = 0.86) | | | | | | | | | | | | |
| **3.6.4 Adalimumab vs. MTX** |  |  |  |  |  |  |  |  |  |  |  |  |
| Papp 2017 | 7 | 77 | 3 | 37 | 22.1% | 1.12 [0.31, 4.09] | **+** | **+** | **+** | **+** | **+** | **+** |
| **Subtotal (95% CI)** |  | **77** |  | **37** | **22.1%** | **1.12 [0.31, 4.09]** |  |  |  |  |  |  |
| Total events | 7 |  | 3 |  |  |  |  |  |  |  |  |  |
| Heterogeneity: Not applicable |  |  |  |  |  |  |  |  |  |  |  |  |
| Test for overall effect: Z = 0.17 (P = 0.86) | | | | | | | | | | | | |

**Total (95% CI)**

Total events

**446**

91

**308**

14

# 100.0%

**2.60 [0.66, 10.25]**

Heterogeneity: Tau² = 1.79; Chi² = 18.67, df = 4 (P = 0.0009); I² = 79% Test for overall effect: Z = 1.36 (P = 0.17)

Test for subgroup differences: Chi² = 4.14, df = 3 (P = 0.25), I² = 27.5%

Risk of bias legend

1. Random sequence generation (selection bias)
2. Allocation concealment (selection bias)
3. Blinding of participants and personnel (performance bias)
4. Blinding of outcome assessment (detection bias)
5. Incomplete outcome data (attrition bias)
6. Selective reporting (reporting bias)
7. Other bias

0.001 0.1 1 10 1000

Control Experimental

| **3.7.1 Etanercept vs.** | **Placebo** |  | | | | | | | | | | |
| --- | --- | --- | --- | --- | --- | --- | --- | --- | --- | --- | --- | --- |
| Paller 2008 | 7 | 106 | 2 | 105 | 22.9% | 3.47 [0.74, 16.30] | **+** | **+** | **+** | **+** | **+** | **+** |
| Siegfried 2010 | 52 | 68 | 10 | 69 | 38.5% | 5.28 [2.93, 9.50] | **?** | **?** | **+** | **+** | **+** | **+** |
| **Subtotal (95% CI)** |  | **174** |  | **174** | **61.4%** | **5.00 [2.89, 8.67]** |  |  |  |  |  |  |
| Total events | 59 |  | 12 |  |  |  |  |  |  |  |  |  |

Heterogeneity: Tau² = 0.00; Chi² = 0.25, df = 1 (P = 0.62); I² = 0% Test for overall effect: Z = 5.74 (P < 0.00001)

# 3.7.2 Secukinumab vs.Etanercept

Bodemer 2021

# Subtotal (95% CI)

Total events

28 80

# 80

28

11 41

# 41

11

38.6%

# 38.6%

1.30 [0.73, 2.35]

# 1.30 [0.73, 2.35]

**? ? + + + +**

Heterogeneity: Not applicable

Test for overall effect: Z = 0.89 (P = 0.37)

# Total (95% CI)

Total events

**254**

87

**215**

23

# 100.0%

**2.80 [0.95, 8.19]**

Heterogeneity: Tau² = 0.69; Chi² = 11.23, df = 2 (P = 0.004); I² = 82% Test for overall effect: Z = 1.87 (P = 0.06)

| **3.7 Nasopharyngitis** | **Experimental** | **Control** | **Risk Ratio** | **Risk Ratio** | **Risk of Bias** |
| --- | --- | --- | --- | --- | --- |
| **Study or Subgroup** | **Events Total** | **Events Total Weight** | **M-H, Random, 95% CI** | **M-H, Random, 95% CI** | **A B C D E F** |

Test for subgroup differences: Chi² = 10.73, df = 1 (P = 0.001), I² = 90.7%

Risk of bias legend

1. Random sequence generation (selection bias)
2. Allocation concealment (selection bias)
3. Blinding of participants and personnel (performance bias)
4. Blinding of outcome assessment (detection bias)
5. Incomplete outcome data (attrition bias)
6. Selective reporting (reporting bias)
7. Other bias

0.001 0.1 1 10 1000

Control Experimental

# Hand fracture

**Experimental Control Risk Ratio**

**Risk Ratio**

**Risk of Bias**

**Study or Subgroup**

**Events**

**Total**

**Events**

**Total**

**Weight**

**M-H, Fixed, 95% CI**

**M-H, Fixed, 95% CI**

**A B C D E F G**

- - 1. **Ustekinumab vs. Placebo**

| Landells 2015 | 20 | 73 | 14 | 37 | 96.5% | 0.72 [0.41, 1.26] | **+ + + + + + +** |
| --- | --- | --- | --- | --- | --- | --- | --- |
| **Subtotal (95% CI)**  Total events | 20 | **73** | 14 | **37** | **96.5%** | **0.72 [0.41, 1.26]** |  |

Heterogeneity: Not applicable

Test for overall effect: Z = 1.14 (P = 0.26)

| **3.8.2 Adalimumab vs. MTX** |  | | | | | | |
| --- | --- | --- | --- | --- | --- | --- | --- |
| Papp 2017 | 1 | 77 | 0 | 37 | 3.5% | 1.46 [0.06, 35.04] | **+ + + + + + +** |
| **Subtotal (95% CI)** |  | **77** |  | **37** | **3.5%** | **1.46 [0.06, 35.04]** |  |
| Total events  Heterogeneity: Not applicable | 1 |  | 0 |  |  |  |  |
| Test for overall effect: Z = 0.23 (P = 0.81) | | | | | | | |
| **Total (95% CI)** |  | **150** |  | **74** | **100.0%** | **0.75 [0.43, 1.30]** | |
| Total events | 21 |  | 14 |  |  |  | |

Heterogeneity: Chi² = 0.18, df = 1 (P = 0.67); I² = 0% Test for overall effect: Z = 1.03 (P = 0.31)

Test for subgroup differences: Chi² = 0.18, df = 1 (P = 0.67), I² = 0%

Risk of bias legend

1. Random sequence generation (selection bias)
2. Allocation concealment (selection bias)
3. Blinding of participants and personnel (performance bias)
4. Blinding of outcome assessment (detection bias)
5. Incomplete outcome data (attrition bias)
6. Selective reporting (reporting bias)
7. Other bias

0.001 0.1 1 10 1000

Control Experimental

| **3.9 Skin eruption** | **Experimental** | | **Control** | |  | **Risk Ratio** | **Risk Ratio** | **Risk of Bias** | |
| --- | --- | --- | --- | --- | --- | --- | --- | --- | --- |
| **Study or Subgroup Events Total**  **3.9.1 Etanercept vs. Placebo** | | | **Events Total** | | **Weight** | **M-H, Random, 95% CI** | **M-H, Random, 95% CI** | **A B C D E F** | |
| Paller 2008 | 16 | 106 | 0 | 105 | 44.0% | 32.69 [1.99, 537.95] | **+ + + + + +** | |  |
| **Subtotal (95% CI)** |  | **106** |  | **105** | **44.0%** | **32.69 [1.99, 537.95]** |  | |  |
| Total events | 16 |  | 0 |  |  |  |  | |  |
| Heterogeneity: Not applicable  Test for overall effect: Z = 2.44 (P = 0.01)  **3.9.2 Secukinumab vs.Etanercept** | | | | | | |  | |  |

Bodemer 2021

# Subtotal (95% CI)

Total events

24 80

# 80

24

10 41

# 41

10

56.0%

# 56.0%

1.23 [0.65, 2.32]

# 1.23 [0.65, 2.32]

**? ? + + + +**

Heterogeneity: Not applicable

Test for overall effect: Z = 0.64 (P = 0.52)

| **Total (95% CI)** |  | **186** |  | **146** | **100.0%** | **5.21 [0.10, 260.11]** |
| --- | --- | --- | --- | --- | --- | --- |
| Total events | 40 |  | 10 |  |  |  |

Heterogeneity: Tau² = 7.01; Chi² = 7.53, df = 1 (P = 0.006); I² = 87% Test for overall effect: Z = 0.83 (P = 0.41)

Test for subgroup differences: Chi² = 5.01, df = 1 (P = 0.03), I² = 80.0%

Risk of bias legend

1. Random sequence generation (selection bias)
2. Allocation concealment (selection bias)
3. Blinding of participants and personnel (performance bias)
4. Blinding of outcome assessment (detection bias)
5. Incomplete outcome data (attrition bias)
6. Selective reporting (reporting bias)
7. Other bias

0.001 0.1 1 10 1000

Control Experimental
