# Supplementary material for: Efficacy and safety of biological agents for the treatment of pediatric patients with psoriasis: A bayesian analysis of six high-quality randomized controlled trials
Source: Front Immunol. 2022 Aug 19;13:896550. doi: 10.3389/fimmu.2022.896550 (PMC9446895; doi:10.3389/fimmu.2022.896550)
Supplement: Supplementary file 6 [file DataSheet_6.docx]

| Node Analysis Results | | | | | | | |
| --- | --- | --- | --- | --- | --- | --- | --- |
| Side | Direct | | Indirect | | Difference | | P>z |
|  | Coef. | Std. Err. | Coef. | Std. Err. | Coef. | Std. Err. |  |
| A C * | 1.699 | 1.052 | 0.499 | 2.257 | 1.199 | 2.545 | 0.637 |
| A D * | 1.277 | 1.060 | 0.078 | 2.261 | 1.199 | 2.545 | 0.637 |
| B C * | 0.232 | 0.990 | 1.432 | 2.341 | -1.199 | 2.545 | 0.637 |
| B D * | -0.189 | 0.998 | 1.010 | 2.344 | -1.199 | 2.545 | 0.637 |
